# PVAylation: precision end-functionalized poly(vinyl alcohol) for site-selective bioconjugation†

**DOI:** 10.1039/d5sc00772k

**Published:** 2025-04-24

**Authors:** Douglas E. Soutar, Ho Fung Mack, Melissa Ligorio, Akalabya Bissoyi, Alexander N. Baker, Matthew I. Gibson

**Affiliations:** a Department of Chemistry, University of Warwick Coventry CV4 7AL UK Matt.gibson@manchester.ac.uk; b Warwick Medical School, University of Warwick Coventry CV4 7AL UK; c Department of Chemistry, University of Manchester Oxford Road Manchester M13 9PL UK; d Manchester Institute of Biotechnology, University of Manchester 131 Princess Street Manchester M1 7DN UK

## Abstract

The (bio)conjugation of polymers onto proteins enhances their pharmacokinetics and stability, most commonly using PEG (polyethylene glycol), but there is a need for alternatives. Poly(vinyl alcohol), PVA, is a water-soluble, biocompatible and environmentally degradable polymer, which also has the unique function of ice recrystallisation inhibition (IRI) which can aid the cryopreservation of biologics. Site-specific PVA bioconjugation (“PVAylation”) is underexplored due to the challenge of obtaining homogenous mono end-functional PVA. Here we show that following deprotection of the acetate (from the precursor poly(vinyl acetate)), the concurrent xanthate end-group reduction leads to a diversity of ambiguous end-groups which prevented precision conjugation. This is overcome by using a photo-catalyzed reduction of the omega-terminal xanthates to C–H, which is orthogonal to active-ester bioconjugation functionality at the alpha-chain terminus, demonstrated by MALDI-TOF mass spectrometry. This strategy enabled the preparation of well-defined mono-functional PVA displaying alkyne, biotin and O^6^-benzylguanine chain-end functionalities, which are each then used for covalent or non-covalent site-specific modification of three model proteins, introduce ice-binding function. These results will enable the synthesis of new bioconjugates containing PVA and be of particular benefit for low-temperature applications.

## Abstract 2

Site-specific bioconjugation of PVA onto proteins "PVAylation" is achieved by precision control of the end groups, as an alternative to PEGylation.

## Graphical abstract

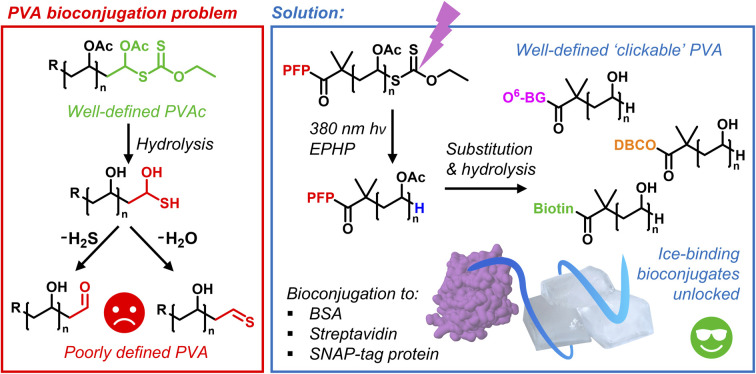

## Introduction

Poly(vinyl alcohol) (PVA) is a water-soluble polymer with a unique combination of properties for a vinyl-derived polymer. It is degradable in the environment by action of microorganisms,^1^ and has low *in vivo* toxicity.^2,3^ PVA is also the most readily accessible, and most active, polymeric ice-recrystallization-inhibitor (IRI).^4–7^ Ice recrystallization is a significant problem during the storage and transport of biologics (or food stuffs), where ice growth can cause mechanical and osmotic damage.^8–10^ PVA has been non-specifically conjugated to lactate dehydrogenase and green fluorescent protein providing stability during several freeze/thaw cycles,^11^ by preventing irreversible aggregation. The addition of PVA into the solution also aided the cryopreservation of several proteins, but required a large molar excess relative to the protein.^5^ Alternatives, such as trehalose-side chain polymers have also been developed for protein stabilization during lyophilization, allowing aggregation-prone biologics such as insulin to be stabilized.^12^ Whilst trehalose-functional polymers are potent and have been proven to be safe in murine models,^13^ they are new-to-human polymers so require extensive testing.

There are no examples, to the best of our knowledge, of the site-specific bioconjugation of well-defined PVA onto proteins. PVA-protein conjugates have, however, been shown to extend blood circulation time *in vivo*.^14^ Site-specific bioconjugation is essential to ensure the conjugation occurs away from active sites (for example, in enzymes) and to enable accurate characterization and hence reproducibility.^15,16^ PEGylation has been widely used but there is a desire to find alternatives to mitigate against anti-PEG antibodies,^17,18^ and several polymers such as polysarcosine^19^ and poly(oxazolines),^20^ as selected examples, have been explored.^21^ These polymers do not bring ice-binding or other advanced cryoprotective properties as bioconjugates and hence require additional excipients to enable storage and transport.

PVA has many synthetic barriers to its bioconjugation which has prevented its wider use despite its appealing properties. Firstly, it has hydroxyl groups along its backbone which can compete with nucleophile/electrophile conjugations. Secondly, PVA is produced by hydrolysis of poly(vinyl esters) such as poly(vinyl acetate) (PVAc), which necessitates any conjugation functionality to be tolerant to the deprotection conditions. Thirdly, VAc is a less-activated monomer with a tendency for chain transfer and head-to-head addition, which makes it challenging to polymerize.^22^ Head-to-head addition has complex effects on ‘living’ polymerization methods, as the stability of the adducts of the two possible chain ends with mediators is not equal.^23^ In both RAFT and ATRP, the dormant form of a growing chain is more stable in the head-to-head variant. In RAFT this leads to accumulation of head-to-head terminated chains, and at low degrees of polymerization, the polymer consists of a mixture of head-to-head and head-to-tail terminated chains. Cobalt mediated polymerization does not have the head-to-head problem, but offers less opportunities for precise end-group functionalization than RAFT.^24^ Advances in RAFT/MADIX polymerization allow facile access to well-defined PVAc.^25^ Particularly promising are photo-RAFT processes which bring advantages in terms of oxygen tolerance and spatio-temporal control over polymerization.^26–28^

The R or Z groups in RAFT derived polymers can be used to enable bioconjugation. For example, amine functional PVA synthesized with a phthalimide R group is deprotected on hydrolysis, but the fate of the Z group is not defined.^29^ Similar strategies have introduced azide^30^ and alkyne^31^ functional R groups. The thiocarbonyl functionality at the ⍵-terminus of RAFT-derived polymers can be reduced to a thiol for disfulfide or thio-Michael additions.^32^ This approach has been used to functionalize oligonucleotides and peptides,^33^ in addition to proteins, when using monomers which result in stable terminal thiols (unlike PVA).^34^ During deprotection of PVAc precursor to obtain PVA, the ⍵-terminal xanthate is cleaved, which to a first approximation would result in a useful thiol-end group. In the case of PVA, however, there is an alcohol on the same carbon as the thiol which is unlikely to be stable, similar to geminal diols. This ambiguity poses significant challenges for subsequent bioconjugation, because the end-group is not ‘pure’ for direct deployment as a conjugation reagent and may interfere with reactivity at the other chain end. With a sufficiently high degree of polymerization, the accumulation of head-to-head chain ends can make the thiol produced upon hydrolysis usable,^35^ however for short PVA lengths the thiol ambiguity problem remains.

Here we introduce a practical and versatile method for the site-specific protein-PVA bioconjugation by precision modification of both chain ends to prevent side reactions. We first demonstrate the ambiguity of PVA end-groups during standard xanthate removal conditions, which prevent straightforward thio-based conjugations. To obtain precision PVAs, pentafluorophenyl (PFP) ester functional PVAc is first prepared by photo-RAFT polymerization using a bismuth oxide catalyst. The xanthate is then selectively reduced to a hydride using a phosphine and UV-light. The PFP ester functionality is not affected by this treatment, allowing it to be displaced by a biotin, alkyne, or O^6^-benzylguanine functional amine to produce ⍺-functional PVAc. Each step is monitored by MALDI-TOF mass spectrometry to assign the end-groups. Following acetate removal, the resulting functional PVAs are deployed for site-specific bioconjugation to model proteins using click, non-covalent, and biocatalytic bioconjugations to demonstrate broad scope. This synthetic approach effectively overcomes the obstacles to PVA being incorporated into protein therapeutics or biocatalysts and will enable downstream applications and wider use of PVAylation.

## Experimental section

### Materials

All chemicals were used as supplied unless otherwise stated. Water used throughout this work was 16 MΩ deionized water from an ELGA chorus 3 water purifier. Reagent grade solvents were used as supplied for reactions and purification. Tetrahydrofuran (THF) 99.5%, dichloromethane (DCM) 99%, ethyl acetate 99%, dimethyl sulfoxide (DMSO) 99.9%, pentane 99.5% and hexane (fraction from petroleum) were purchased from Fisher Scientific. Ethanol absolute was purchased from VWR chemicals. Methanol 99.8%, isopropyl alcohol 99.8%, UPHLC grade acetonitrile (for MALDI) and 99.9% anhydrous inhibitor free THF (for MALDI) were purchased from Sigma Aldrich.

Vinyl acetate >99%, (3–20 ppm hydroquinone as inhibitor) was purchased from Sigma Aldrich and passed over a short plug of neutral alumina before polymerization to remove inhibitor. Potassium ethyl xanthogenate, 2-bromo-2-methyl propionic acid 98%, bismuth(iii) oxide powder 99.999% trace metals basis, 1-ethylpiperidine hypophosphite, hydrazine hydrate 50–60% reagent grade, 5.4 M sodium methoxide in methanol, dibenzocyclooctyne-amine, DCTB 98%, 2,5-DHB 99%, sodium trifluoroacetate 99%, and potassium trifluoroacetate 99% were purchased from Sigma Aldrich. 1-ethyl-3-(3-dimethylaminopropyl)carbodiimide hydrochloride (EDC) was purchased from Carbosynth. Pentafluorophenol 99% and trifluoroacetic acid 99% were purchased from ABCR. Sulfuric acid 20%, magnesium sulfate were purchased from Fisher Scientific. Benzylamine 99% was purchased from Acros Organics. EZ-Link™ pentylamine-biotin was purchased from Thermo Scientific. 6-((4-(aminomethyl)benzyl)oxy)-7*H*-purin-2-amine (BG-NH_2_) was purchased from Fluorochem.

CDCl_3_, 0.03% TMS, was purchased from Apollo Scientific. CDCl_3_ (no TMS), DMSO-d_6_, and D_2_O were purchased from Sigma Aldrich. Octet® Streptavidin (SA) biosensors were purchased from Sartorius.

### Physical and analytical methods

#### NMR spectroscopy

^1^H-NMR and ^13^C-NMR spectra were recorded at either 400 MHz or 300 MHz on a Bruker Avance III spectrometer, using chloroform-d (CDCl_3_), DMSO-d_6_ or D_2_O as the solvent. Chemical shifts of protons are reported as *δ* in parts per million (ppm) are relative the solvent residual peak (CHCl_3_*δ* = 7.264 ppm, H_2_O *δ* = 4.79 ppm, DMSO *δ* = 2.50 ppm).

#### Size exclusion chromatography

SEC analysis of poly(vinyl acetate) polymers was performed on an Agilent Technologies 1260 Infinity MDS instrument equipped with differential refractive index (DRI), light scattering (LS) and viscometry (VS) detectors, using 2× PLgel Mixed-D columns. The solvent was DMF with 5 mM NH_4_BF_4_. The run time was 45 minutes with flow rate 1 ml min^−1^. Polymer samples were dissolved in eluant before filtering with a PTFE 0.22 μm filter. Number average molecular weights (*M*_n_), weight average molecular weights (*M*_w_) and dispersities (*Đ* = *M*_w_/*M*_n_) were determined by conventional calibration against poly(methyl methacrylate) standards using Agilent SEC software.

#### MALDI-TOF-MS

MALDI-TOF-MS was performed using a Bruker Autoflex Speed, with an MTP 384 ground steel target plate. For PVAc samples, a saturated solution of DCTB in THF was prepared. A solution of 2 mg ml^−1^ KTFA was prepared in THF. A solution of the PVAc sample in THF at 1 mg ml^−1^ was prepared. PVAc solution was mixed with KTFA solution at a ratio of 1 : 5, before spotting 0.25 μl of this mixture onto a pre-dried 0.25 μl DCTB spot.

For PVA samples, a ‘TA30’ solution was prepared by mixing 7 parts TFA solution (0.01% trifluoracetic acid in water) with 3 parts acetonitrile. PVA samples were dissolved in TA30 at concentration 2 mg ml^−1^. A saturated solution of 2,5-DHB in TA30 was prepared. A 2 mg ml^−1^ solution of KTFA in TA30 was prepared. The sample, KTFA and 2,5-DHB solutions were mixed at a ratio of 1 : 1 : 10 respectively and 1 μl of this solution was spotted onto the plate. The plate was dried under an extractor hood at room temperature. KTFA was used as a cationization agent because this reliably produced a single distribution rather than various H+, Na+, K+ adducts.

Splat, biolayer interferometry and other associated techniques are detailed in the ESI.†

### Synthetic procedures

Xanthate and polymer synthesis methods are detailed in the ESI† as well as NMR spectra, SEC data, and methods for MALDI-TOF MS, BLI, SDS-PAGE and IRI assay.

#### Photoinduced xanthate removal from PVAc

Removal of xanthates was performed in a Heptatochem photoredox box using a 18 W 380 nm LED lamp, model HCK1012-01-013 with specified relative irradiance of 8 mW cm^−2^. 8 ml glass vials with a rubber septum were used, which when filled have approximately 6 cm^2^ surface area, with an absolute irradiance per vial of 50 mW.

#### PVAc_19_-H

126 mg PVAc_19_-Xan (approx. 1850 g mol^−1^, 0.068 mmol, 1 eq.) was dissolved in 1 ml methanol, and added to a vial containing 385 mg (2.15 mmol, 31.6 eq.) 1-ethylpiperidine hypophosphite (EPHP). The solution was degassed by nitrogen sparging for 10 minutes, then stirred and exposed to 380 nm light for 3 hours. The polymer was precipitated in water, then redissolved in methanol and precipitated in water again. After shortly drying under vacuum, the polymer was precipitated from THF in hexane twice, then dried under vacuum yielding 90 mg modified PVAc.

#### PFP-PVAc_23_-H

1.04 g (approx. 2400 g mol^−1^ 0.43 mmol, 1 eq.) PFP-PVAc_23_-Xan was dissolved in 3 ml methanol. 1.34 g (179.20 g mol^−1^ 7.47 mmol, 17 eq.) EPHP was dissolved in 3 ml methanol. Both solutions were combined in 8 ml vial, a magnetic stir bar was added, and rubber septum fitted, then the solution was degassed by nitrogen sparging for 20 minutes. The solution was then exposed to 380 nm light in the photoreactor with moderate stirring. A sample was used for ^1^H NMR after 5 hours which confirmed the reaction was complete, and exposure was stopped at 6 hours. The PVAc was precipitated in water, rinsed with pentane, centrifuged, then precipitated from THF in pentane twice. The product was dried under vacuum for 2 hours, yielding 0.954 g modified PVAc.

#### Synthesis of Bzl-PVAc_23_

54.9 mg (0.024 mmol) PFP-PVAc_23_-H was dissolved in 1 ml dioxane. 13 μl benzylamine (∼12.7 mg, 0.12 mmol, 5 eq.) was added. The solution was heated to 50 °C for 2 hours with gentle stirring in a sealed vessel. ^19^F NMR of the reaction mixture showed complete transformation of the PFP-ester to free pentafluorophenol. After 3.5 hours, the polymer was precipitated in 6 ml water, then washed several times with pentane, and dried under vacuum, leaving 55 mg Bzl-PVAc_23_. 1.5 mg product was dissolved in CDCl_3_ for NMR analysis. The remaining polymer was immediately deacetylated.

#### Deacetylation of Bzl-PVAc_23_ to Bzl-PVA_23_

50 mg Bzl-PVAc_23_ was dissolved in 160 μl methanol, then 100 μl of 50–60% hydrazine hydrate solution was added. The solution was stirred at room temperature for 16 hours. The reaction mixture was precipitated in 1.5 ml isopropyl alcohol, then the solid was washed with 1 ml isopropyl alcohol, washed with 1 ml pentane twice, then dried under vacuum.

#### Synthesis of biotin-PVA_23_-H

4.3 mg pentylamine-biotin and 19.7 mg PFP-PVAc_23_-H were dissolved in 500 μl DMF, and heated to 50 °C. After 21 hours, a sample of the reaction mixture was taken for ^19^F NMR which showed complete hydrolysis of the PFP ester. The PVAc was precipitated in 1.5 ml water and after centrifugation was dried under vacuum. The entire sample was dissolved in 650 μl CDCl_3_ for NMR experiments, after which the solvent was removed under reduced pressure. The PVAc was redissolved in 100 μl methanol, then 50 μl hydrazine hydrate solution was added. The solution was stirred for 3 hours at 50 °C, then the polymer was precipitated in 1.5 ml isopropyl alcohol, cooled with liquid nitrogen to encourage complete precipitation then centrifuged and dried under vacuum. 3.4 mg biotin-PVA recovered, and analyzed by ^1^H NMR in D_2_O, and MALDI-TOF MS.

#### Synthesis of DBCO-PVA_23_

26.1 mg PFP-PVAc_23_-H dissolved in 1 ml DMF. 4.1 mg DBCO amine was added. The mixture was agitated on a roller until completely dissolved, then heated to 50 °C and stirred for 2 hours 30 minutes. The DMF was removed under vacuum. The product was dissolved in 1 ml methanol, cooled in an ice bath, and 100 μl 5.4 M methanolic sodium methoxide solution was added. The solution was stirred for 10 minutes, then stirred at 25 °C for 1 hour. The product was precipitated in isopropyl alcohol cooled with liquid nitrogen, and centrifuged. The pellet was redissolved in water 1 ml, washed with ethyl acetate 1 ml three times, and lyophilized.

#### Synthesis of BG-PVA_23_

176 mg PFP-PVAc_23_-H and 32.5 mg BG-NH_2_ were dissolved in 6 ml DMF, and stirred at 50 °C for 2 hours, at which point a sample was taken for ^19^F NMR which showed an incomplete reaction. A further 39 mg BG-NH_2_ was added, and the reaction stirred for a further 4 hours, after which ^19^F NMR showed completion. The DMF was removed at 50 °C under vacuum. 6 ml methanol was added, the solution was cooled to 0 °C and 600 μl 5.4 M methanolic sodium methoxide solution was added. The reaction was stirred for 16 hours. The solution was neutralized by adding 3 ml water and amberlite IR 120H + resin. Then the solution was precipitated in cold acetone and centrifuged. The pellet was washed with methanol and dried under vacuum. 31 mg BG-PVA was recovered.

#### Deacetylation of unmodified PFP-PVAc_23_-H

50.4 mg PFP-PVAc_23_-H was dissolved in 200 μl methanol, and 100 μl hydrazine hydrate 50–60% solution was added. The solution was stirred at 50 °C for 4 hours then precipitated in IPA, redissolved in 100 μl water, precipitated in 2 ml IPA and dried under vacuum. 20 mg PVA was recovered.

## Results and discussion

**Fig. 1:**
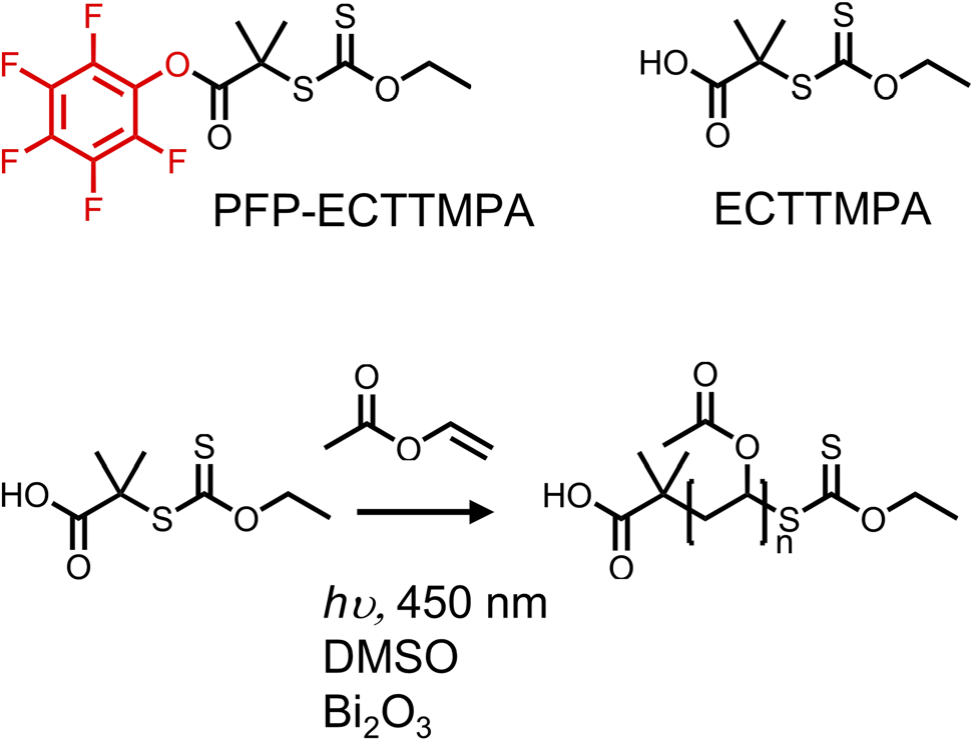
PET-RAFT of vinyl acetate with bismuth oxide as catalyst, under 450 nm (blue) light irradiation, using either ECTTMPA or PFP-ECTTMPA as the CTA.

The primary aim of this study was to develop an accessible yet precise synthetic methodology for site-selective protein bioconjugation of poly(vinyl alcohol) (PVA) at a single chain end. This would enable its wider exploration as an alternative to other water-soluble polymers (such as PEG) and enable PVA's advanced functions such as ice-binding to be integrated into biohybrid conjugates. First, to synthesise poly(vinyl acetate) (PVAc) with high end-group fidelity, PET-RAFT/MADIX was employed, using 2-((ethoxycarbonothioyl)thio)-2-methylpropanoic acid, (ECTTMPA) and pentafluorophenyl 2-((ethoxycarbonothioyl)thio)-2-methylpropanoate (PFP-ECTTMPA) (Fig. 1). VAc is a less activated monomer with a tendency for side reactions including chain transfer, which makes it difficult to achieve high conversion without higher initiator concentrations when conventional RAFT polymerization is used. Photoiniferter and PET-RAFT polymerizations can produce polymers with higher end-group fidelity than those produced using thermal initiators, due to the absence initiator-derived chains.^36^ Several photocatalysts such as tris(2-phenylpyridine)iridium have been reported,^37^ but bismuth oxide was chosen here, due to ease of removal (heterogeneous catalyst), which combined with the lack of need for deoxygenation^27,38^ makes this method accessible. The mechanism of this method involves reduction of molecular oxygen by the photocatalyst such that it no longer inhibits propagation, but trace oxygen species also play a role in the catalytic cycle.^39^ Using this polymerization system, PVAc was prepared using two chain transfer agents (Fig. 1), reaching at least 75% conversion overnight, with dispersity below 1.2 (Table 1). Relatively low DPs were targeted to enable full characterization throughout.

**Table 1:** PVAc synthesized using Bi_2_O_3_ catalyzed PET-RAFT

Polymer	[*M*]:[CTA]	CTA	Conversion	Time	*M*_n_, *Đ* (SEC)^a^	DP, (NMR)^b^	DP (theoretical)^c^
PVAc_19_-Xan	25	ECTTMPA	76%	18h	3800, 1.17	19	19
PFP-PVAc_23_-Xan	25	PFP-ECTTMPA	85%	16h	3900, 1.19	23	21

a SEC was performed in DMF verses poly(methyl methacrylate) standards.

b Calculated using the ^1^H NMR integration of the xanthate O-ethyl CH_2_ compared to the PVAc backbone integrations.

c Conversion multiplied by [M]:[CTA].

**Fig. 2:**
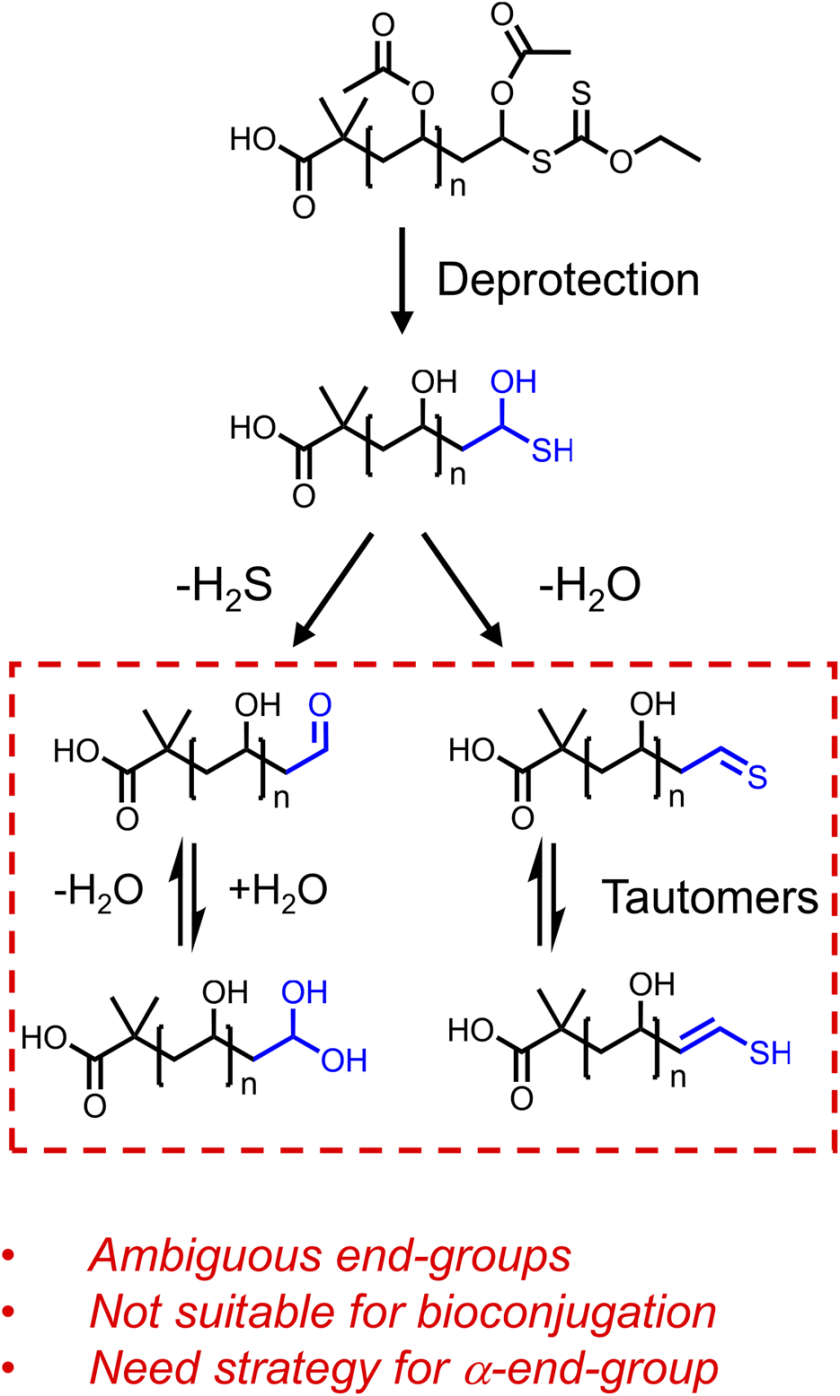
End-group ambiguity following removal of the acetate groups from PVAc. The possible pathways are shown based on H_2_O or H_2_S loss supported by MALDI-MS evidence (ESI†).

We first attempted to use ECTTMPA derived PVAc to produce ω-thiol-terminated polymers suitable for thiol-ene reactions with maleimides. However, we observed that the conversion to the succinimide product was low and irreproducible. MALDI-MS evidence revealed that a thioaldehyde (or a –CHCH–SH functionality) is the main ω-end-group present after deacetylation of PVAc_19_-Xan (Fig. S18†), suggesting water is eliminated from the ω-chain end following deacetylation (Fig. 2). There is likely a complex mixture of products and the equilibrium will shift with the preparation and analysis conditions, and hence results in barriers to precision bioconjugation. We also attempted to capture the thiol *in situ* during a ‘one-pot’ thiol-ene reaction of PVAc_19_-Xan with benzyl acrylamide, which did not reliably produce the thiol-ene product (Fig. S21†), supporting the hypothesis that the thiol adjacent to an acetate may also be unstable or engaging in side-reactions. This highlights a key barrier to the deployment of RAFT-derived PVA as a bioconjugation reagent, as the terminal sulfur containing species may interfere with any conjugation chemistry at the other chain end, while not being ‘pure’ enough itself to be used. The aim of this study was not to quantify these equilibrium products but to develop the tools to prevent them being a problem, making RAFT-derived PVAc a viable route to end-functional poly(vinyl alcohol).

To address the above problem, our proposed synthetic strategy was to remove the xanthate ω-end-group before removal of the acetate groups, to prevent the ⍵-terminal ambiguity. We could then exploit a pentafluorophenol (PFP) ester group at the α-terminus of the PVAc to install diverse bioconjugation functionalities. A key consideration here, is that any methodology must retain the PFP ester at the ⍺-terminus, to subsequently install biorthogonal functional groups which themselves would not be tolerant to radical polymerization conditions.

**Fig. 3:**
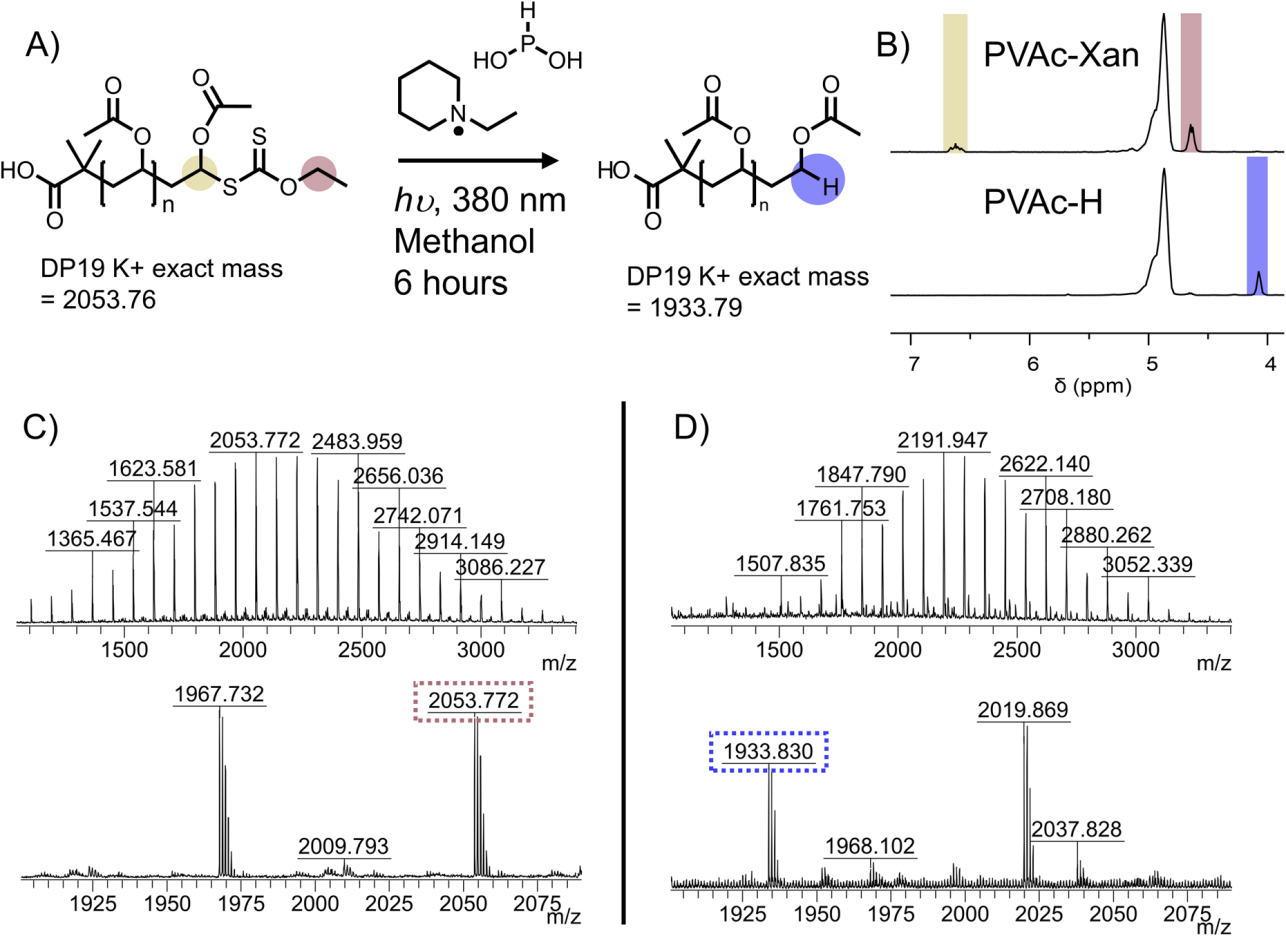
Photocatalytic reduction of xanthate to C–H. (A) Reaction scheme. (B) Region of ^1^H NMR showing disappearance of C**H**_2_ of xanthate at 4.64 ppm and appearance of terminal C(OAc)**H**_2_ at 4.08 ppm (Fig. S9† for full spectra). MALDI-MS mass spectra for (C) PVAc_19_-Xan and (D) PVAc_19_-H. Peaks correspond to K^+^ adducts of the polymers.

Treatment with UV light and *N*-ethylpiperidine hypophosphite was identified as a suitable method for reduction of a xanthate to terminal hydride while avoiding high temperatures which could degrade the activated ester (Fig. 3A).^40^ Photoexcitation of the xanthate ester leads to cleavage at the C–S bond, forming a thiyl radical and a polymer radical. The EPHP then acts as a hydrogen atom donor, capping the polymer. The thiyl radical likely forms ethyl xanthic acid as a side product. We first tested the method on PVAc-Xan_19_ (Table 1), with an average degree of polymerization of 19 which allows end-groups to be resolved in ^1^H NMR. Following 3 hours irradiation at 380 nm in the presence of approximately 30-fold molar excess of EPHP, quantitative removal of the xanthate C**H**_2_ peak at 4.64 ppm in ^1^H NMR was observed. The peak at 6.62 ppm corresponding to the terminal C**H**(OAc)Xan was also removed. A new peak attributable to C(OAc)**H**_**2**_ appeared at 4.05 ppm (Fig. 3B). MALDI-TOF MS revealed a clear shift in the observed molecular weight, corresponding to replacement of the xanthate with hydride across the entire distribution (Fig. 3C and D).

**Fig. 4:**
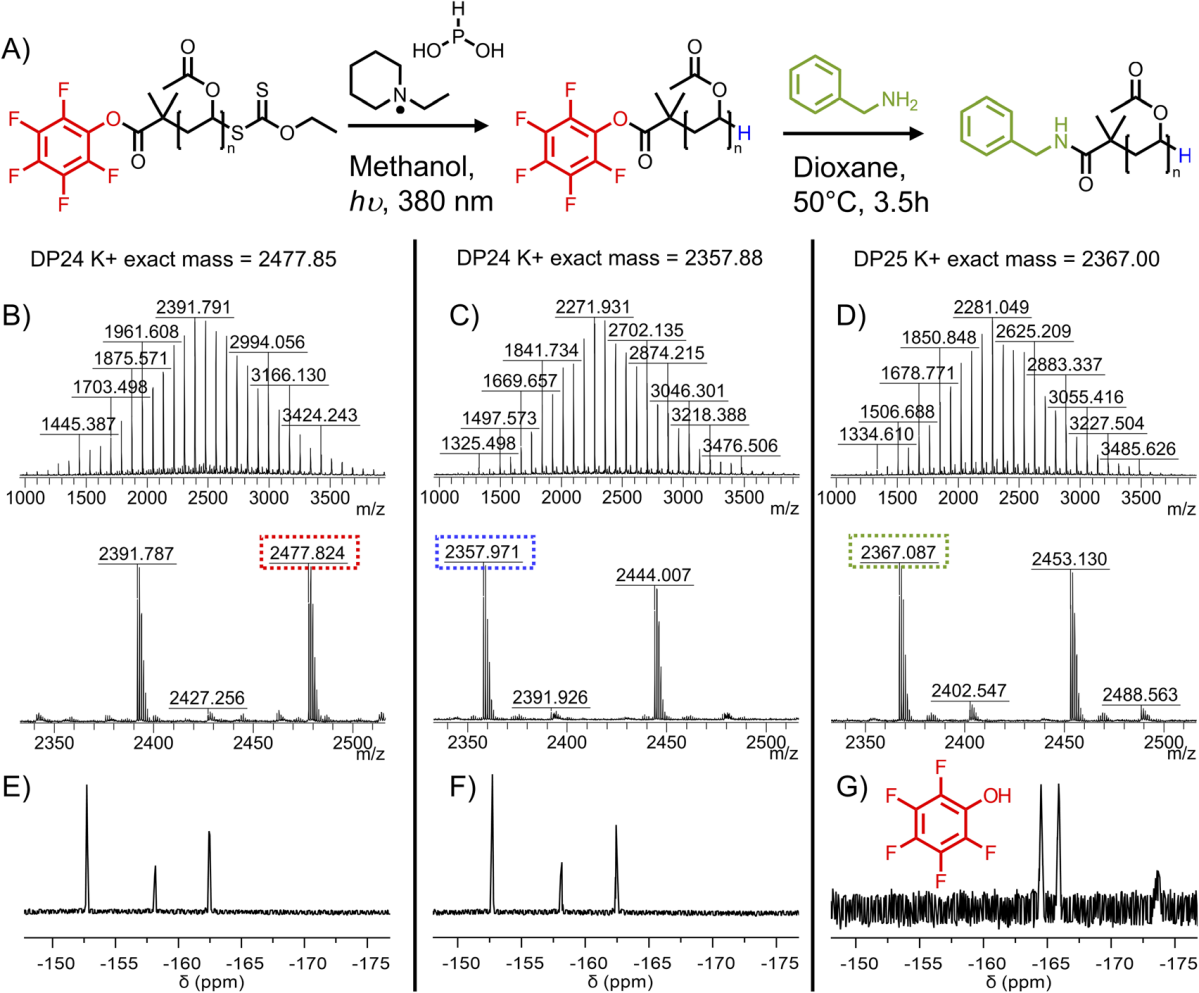
H(A) Reaction scheme for transformation of PFP-PVAc-Xan to Bzl-PVAc: MALDI-TOF MS spectra showing K^+^ adducts of polymers for; (B) PFP-PVAc_23_-Xan; (C) PFP-PVAc_23_-H; (D) Bzl-PVAc_23_-H ^19^F NMR of the polymers (E) PFP-PVAc_23_-Xan; (F) PFP-PVAc_23_-H; (G) Bzl-PVAc_23_-H.

The end-group cleavage method was then applied to PFP-PVAc_23_-Xan, with the aim of retaining the PFP group. PFP-PVAc_23_-Xan was reacted with 17 equivalents EPHP for 6 hours. MALDI-MS confirmed xanthate removal (Fig. 4B and C) with complete retention of the PFP end group, supported by ^19^F NMR evidence showing no new fluorine environments (Fig. 4E and F). Comparison of molecular weight distributions from SEC revealed a minimal amount of higher molecular weight material after EPHP treatment (Fig. S24†), which was not observable by MALDI-TOF MS.

To confirm the availability of the ⍺-terminal PFP for substitution, the isolated PFP-PVAc_23_-H was reacted with an excess of benzylamine (to provide a handle easily quantified by ^1^H NMR). After 2 hours, ^19^F NMR of the reaction mixture showed complete removal of the PFP-ester to release pentafluorophenol (Fig. 4G). The purified polymer was analysed by ^1^H NMR (Fig. S14†) and by MALDI-TOF MS (Fig. 4D) showing the expected masses consistent with installation of the benzyl group.

**Fig. 5:**
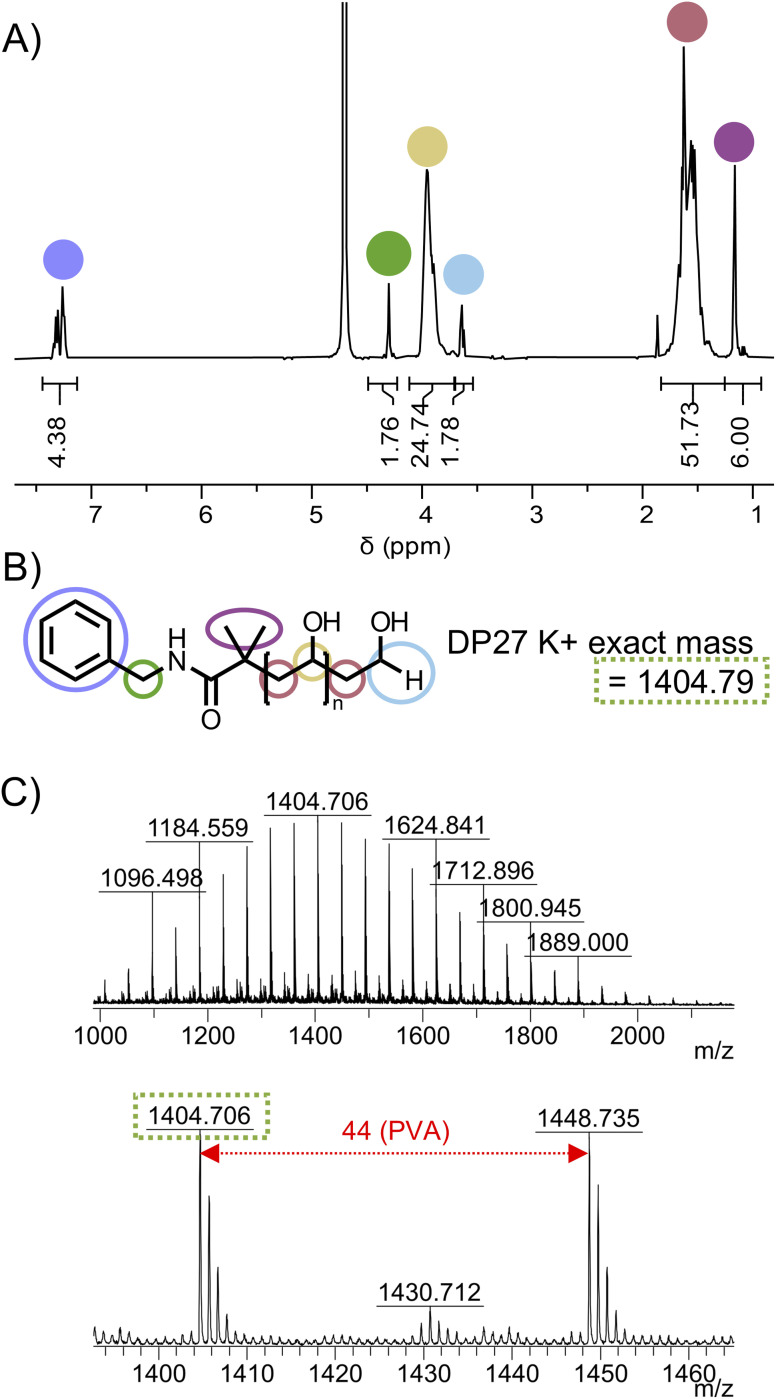
Homogeneous Bzl-PVA_23_ produced by hydrolysis of Bzl-PVAc_23_ (A) ^1^H NMR spectra in D_2_O showing installation of the benzyl group into PVA; (B) structure and exact mass for K^+^ ion of Bzl-PVA_23_. (C) MALDI-TOF mass spectrum of Bzl-PVA_23_.

Benzylamide-PVAc_23_-H was dissolved in methanol and treated with aqueous hydrazine hydrate solution to remove the acetate groups to produce benzylamide modified PVA, Bzl-PVA_23_, which was purified by precipitation. The structure for Bzl-PVA_23_ was confirmed using ^1^H NMR (Fig. S15†) and MALDI-TOF MS (Fig. 5) demonstrating homogeneous PVA with precisely defined end-groups was obtained by this two-step functionalisation protocol.

**Fig. 6:**
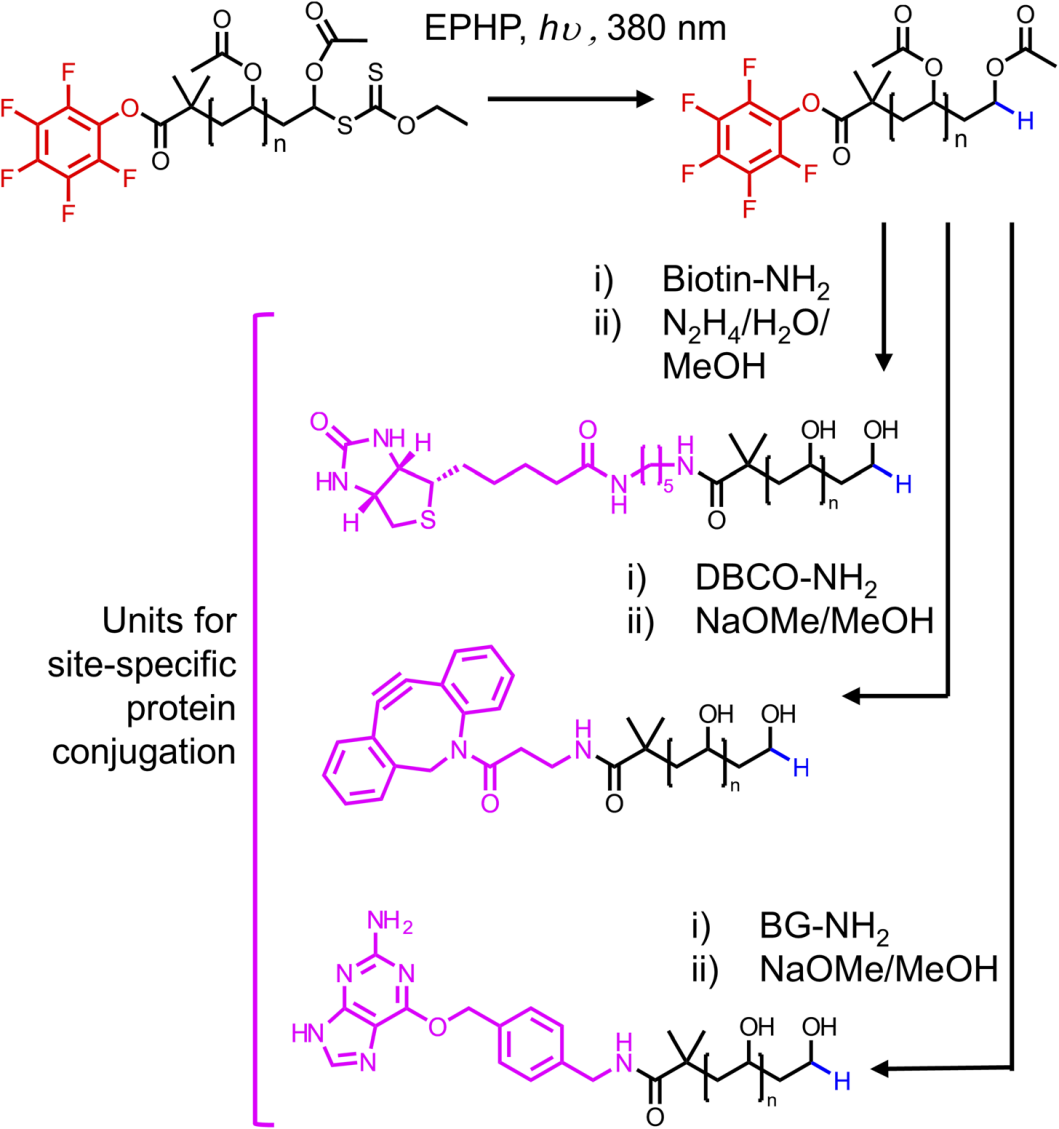
Synthetic routes to obtain ⍺-functional PVA suitable for bioorthogonal conjugation to proteins.

**Fig. 7:**
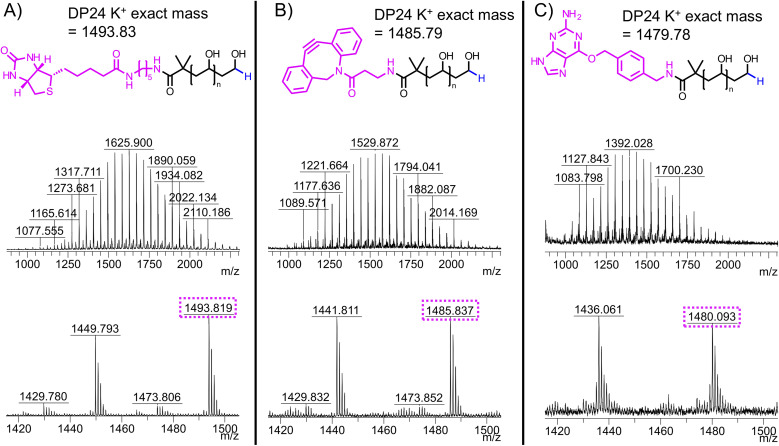
Structures and MALDI-TOF mass spectra for (A) biotin-PVA, (B) DBCO-PVA and (C) BG-PVA.

Having determined a route to *α*-functional PVA, we wanted to explore the installation of diverse functionalities suitable for bioconjugation. We chose biotin, dibenzocyclooctyne (DBCO), and O^6^-benzylguanine. For each substituent, we used their respective amine to substitute the PFP-PVAc-H in a similar manner to benzylamine. The routes to PVA differed in the deprotection method, where for DBCO and O^6^-benzylguanine methanolic NaOMe was used to avoid side reactions with hydrazine (Fig. 6). Synthesis of each substituted PVA was confirmed by MALDI-TOF MS (Fig. 7). Substitution of biotin-PVAc_23_ and retention of the biotin after deacetylation was also confirmed by ^1^H NMR (Fig. S16 and S17†).

**Fig. 8:**
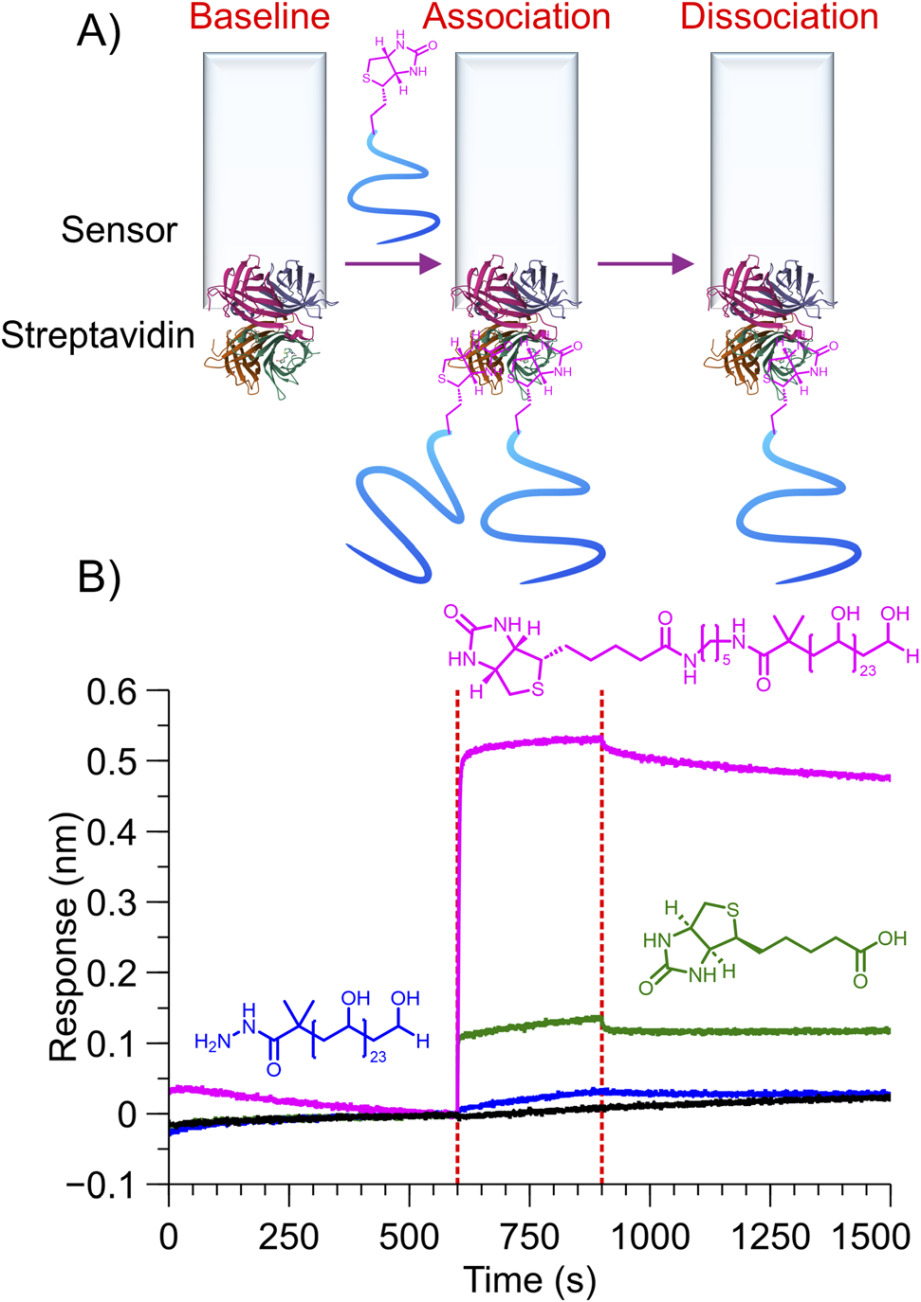
BLI (biolayer interferometry) to assess capture of biotin-PVA_23_ to streptavidin-coated sensors. (A) Schematic of the interaction and capture of biotin-PVA_23_ onto the sensor. (B) BLI output showing specific capture of the biotin-PVA (magenta), verses unmodified PVA (blue), biotin alone (green) and buffer alone (black).

Biotin engages in strong non-covalent interactions with streptavidin.^41^ Biolayer interferometry (BLI) was used to demonstrate the selective interaction between the biotinylated PVA and streptavidin. BLI is a surface-sensitive technique, with an increase in signal output corresponding to mass captured. Commercial streptavidin-coated BLI sensors were exposed to biotin alone, biotin-PVA_23_, or PVA_23_ at the same molar concentration. The biotin-PVA gave a large irreversible signal confirming capture, while the biotin alone gave a small signal (Fig. 8). Unmodified PVA and buffer controls showed no signal, confirming the selective bioconjugation of biotin-PVA.

**Fig. 9:**
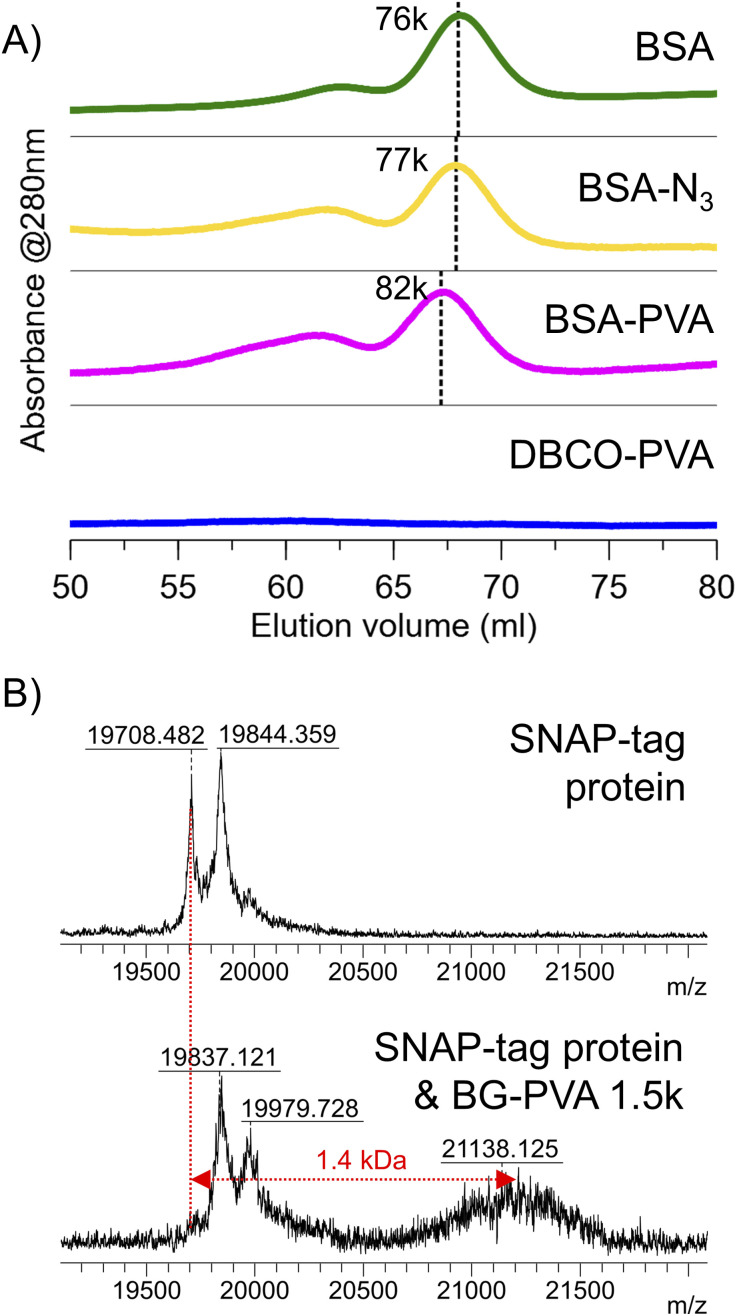
Bioconjugations of DBCO-PVA_23_ and BG-PVA_23_. (A) Extrapolated molecular weight distributions from FPLC to confirm the conjugation of DBCO-PVA_23_-H to BSA-azide. (B) MALDI-MS spectra of the 1^+^ charge state of snap protein compared to a mixture of snap protein and BG-PVA_23_.

Encouraged by the above, an azide-alkyne cycloaddition (SPAAC) ‘click’ reaction was used to conjugate DBCO-PVA_23_ with mono-azido bovine serum albumin (BSA). An excess (>100 molar equivalents) of DBCO-PVA_23_ was reacted at room temperature overnight in phosphate buffer with BSA-azide at a 0.25 mg.ml^−1^. The conjugation was confirmed by a shift in the molecular weight visible by both FPLC (Fig. 9A) and SDS-PAGE (Fig. S26†). By both methods, the shift in retention volume/retention factor corresponded to approximately 2 kDa, which is consistent with successful bioconjugation. Using the ‘splat’ assay,^4,42^ the PVA_23_-BSA conjugate was shown to retain ice recrystallisation inhibition (when corrected to total PVA concentration) demonstrating that bioconjugation does not negatively impact PVA's additional functionality (Fig. S25†).

Next, the conjugation of O^6^-benzylguanine-PVA to SNAP-tag protein^43^ was confirmed by MALDI-TOF MS (Fig. 9B). After reaction of 4 equivalents PVA with snap tag at 37 °C for 30 minutes in PBS with DTT, the mass spectrum shows a new species at 21.1 kDa, adjacent to snap protein at 19.7 kDa. The new peak was also visible (with higher resolution) in the 2+ and 3+ charge states (Fig. S27†). While the conjugation was not quantitative, it demonstrates the single addition. We suspect a different DTT concentration or use of an alternative reducing agent in the buffer may improve the extent of conjugation since the remaining snap protein is mainly the form reduced by DTT, but we did not optimize further as the aim was to demonstrate the installation and bioavailability of the chain-end functionality for bioconjugation.

**Fig. 10:**
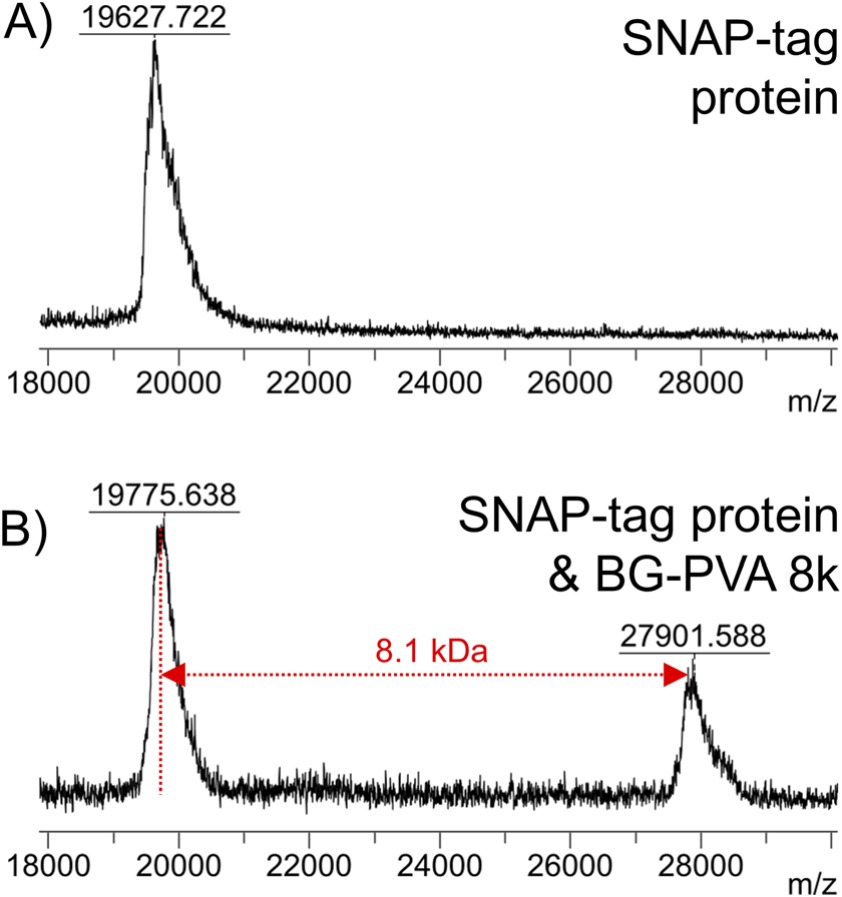
MALDI-TOF mass spectra of (A) SNAP-tag protein (A) and reaction mixture of snap tag protein + BG-PVA_110_ (B).

To demonstrate the versatility of this method, SNAP-tag conjugation with a longer PVA (BG-PVA_110_) was undertaken. It was observed that the EPHP treatment step required longer (24 hours) UV exposure time to remove the xanthate fully, which in methanol resulted in partial hydrolysis of the PFP-ester. Changing the solvent to 50 : 50 mixture of THF : IPA allowed complete xanthate removal and retention of the PFP-ester (Fig. S30†). Following addition of O^6^-benzylguanine and hydrolysis, this polymer was successful conjugated to the SNAP-tag protein, shown by MALDI-TOF MS (Fig. 10).

## Conclusions

In conclusion, we have demonstrated a synthetic route for the site-specific bioconjugation of poly(vinyl alcohol), PVA, onto proteins to enable this versatile, but challenging, polymer to be integrated into bioconjugates. Firstly, a photochemical route was used to reduce the RAFT/MADIX-derived xanthate end-group to a C–H bond of the PVA precursor, PVAc, using *N*-ethylpiperidine hypophosphite. This step prevents the generation of ambiguous (sulfur containing) ω end-groups at the chain-end of PVA would otherwise interfere with conjugations, and are themselves unsuitable for precision conjugation. The photoredox strategy was shown to be compatible with pentafluorophenyl functional ⍺ chain ends, which were subsequently available to install the required bioorthogonally reactive units for protein conjugation, which themselves are not always tolerant to controlled radical polymerization conditions. Following complete hydrolysis of acetates of the modified PVAc, precision PVA was obtained bearing alkyne, biotin and benzylguanine end-groups, for strain promoted click, streptavidin capture or ‘SNAP’ tag bioconjugations, respectively. Bioconjugation was demonstrated using a range of methods including MALDI-TOF MS, fast protein liquid chromatography (FPLC), gel electrophoresis and biolayer interferometry. The PVA-BSA conjugate retained the ice-binding and ice-recrystallisation inhibition activity of PVA, which is not introduced by PEG or other water-soluble biocompatible polymers. PVA bioconjugation is appealing as an alternative to the standard PEGylation, bringing advanced functionality including (environmental) biodegradability and ice binding/recrystallisation inhibition functionality, not found in most synthetic polymers. Future work will explore bioconjugates of PVA, particularly for intrinsically cryo-stable bioconjugates with resistance to protein degradation.

## Data availability

All data is within the supporting information.

## Author contributions

DES, ML, ANB, HFM conducted experiments. DES and MIG devised experiments and analyzed the data. MIG directed the research. MIG and ANB supervised DES. All authors contributed to writing the manuscript.

## Conflicts of interest

There are no conflicts to declare.

## Supplementary Material

SC-016-D5SC00772K-s001
