# Supplementary material for: PVAylation: precision end-functionalized poly(vinyl alcohol) for site-selective bioconjugation
Source: Chem Sci. 2025 Apr 24;16(21):9264–75. doi: 10.1039/d5sc00772k (PMC12019296; doi:10.1039/d5sc00772k)
Supplement: SC-016-D5SC00772K-s001 [file SC-016-D5SC00772K-s001.pdf]

Supplementary Information (SI) for Chemical Science.
This journal is © The Royal Society of Chemistry 2025

Supporting Information For

PVAylation: Precision End-Functionalized Poly(vinyl alcohol) for Site-Selective Bioconjugation

Douglas E. Soutar[a], Ho Fung Mack[c,d] Melissa Ligorio,[a] Akalabya Bissoyi[c,d], Alexander N. Baker[a] and Matthew I. Gibson[a,b,c,d *]

[a)] Department of Chemistry, University of Warwick, Coventry, CV4 7AL, United Kingdom

[b)] Warwick Medical School, University of Warwick, Coventry, CV4 7AL, United Kingdom

[c)] Department of Chemistry, University of Manchester, Oxford Road, Manchester, M13 9PL, UK

[d)] Manchester Institute of Biotechnology, University of Manchester, 131 Princess Street, Manchester, M1 7DN, UK

Corresponding Author Contact. Matt.gibson@manchester.ac.uk

Experimental Section

Materials

All chemicals were used as supplied unless otherwise stated. Water used throughout this work was 16 MΩ deionized water from an ELGA chorus 3 water purifier. Reagent grade solvents were used as supplied for reactions and purification. Tetrahydrofuran (THF) 99.5%, dichloromethane (DCM) 99%, ethyl acetate 99%, dimethyl sulfoxide (DMSO) 99.9%, pentane 99.5% and hexane (fraction from petroleum) were purchased from Fisher Scientific. Ethanol absolute was purchased from VWR chemicals. Methanol 99.8%, isopropyl alcohol 99.8%, UPHLC grade acetonitrile (for MALDI) and 99.9% anhydrous inhibitor free THF (for MALDI) were purchased from Sigma Aldrich.

Vinyl acetate >99%, (3-20 ppm hydroquinone as inhibitor) was purchased from Sigma Aldrich and passed over a short plug of neutral alumina before polymerization to remove inhibitor. Potassium ethyl xanthogenate, 2-bromo-2-methyl propionic acid, bismuth(III) oxide powder 99.999% trace metals basis, 1-ethylpiperidine hypophosphite, hydrazine hydrate 50-60% reagent grade, 5.4 M sodium methoxide in methanol, DCTB 98%, super-DHB 99%, sodium trifluoroacetate 99%, and potassium trifluoroacetate 99% were purchased from Sigma Aldrich. 1-ethyl-3-(3-dimethylaminopropyl)carbodiimide hydrochloride (EDC) was purchased from Carbosynth. Pentafluorophenol 99% and trifluoroacetic acid 99% were purchased from ABCR. Sulfuric acid 20% and magnesium sulfate were purchased from Fisher Scientific. Benzylamine 99% was purchased from Acros Organics. EZ-Link™ pentylamine-biotin was purchased from Thermo Scientific.

$CDCl_3$, 0.03% TMS, was purchased from Apollo Scientific. $CDCl_3$ (no TMS), DMSO-D_6, and D_2O were purchased from Sigma Aldrich. Octet® Streptavidin (SA) biosensors were purchased from Sartorius.

Physical and Analytical Methods

NMR Spectroscopy

^{1}H-NMR and ^{13}C-NMR spectra were recorded at either 400 MHz or 300 MHz on a Bruker Avance III spectrometer, using chloroform-d ($CDCl_3$), DMSO-d6 or D_2O as the solvent. Chemical shifts of protons are reported as δ in parts per million (ppm) are relative the solvent residual peak ($CHCl_3$ δ = 7.264 ppm, H_2O δ = 4.79 ppm, DMSO δ = 2.50 ppm).

Size Exclusion Chromatography

SEC analysis of poly(vinyl acetate) polymers was performed on an Agilent Technologies 1260 Infinity MDS instrument equipped with differential refractive index (DRI), light scattering (LS) and viscometry (VS) detectors, using 2x PLgel Mixed-D columns DMF with 5 mM NH_4BF_4. The run time was 45 minutes with flow rate 1 mL/min. Polymer samples were dissolved in eluant before filtering with a PTFE 0.22 µm filter. Number average molecular weights (Mn), weight average molecular weights (Mw) and dispersities (Đ = Mw/Mn) were determined by conventional calibration against poly(methyl methacrylate) standards using Agilent SEC/SEC software.

MALDI-TOF-MS

MALDI-TOF-MS was performed using a Bruker Autoflex Speed, with an MTP 384 ground steel target plate. For PVAc samples, a saturated solution of DCTB in THF was prepared. A solution of 2 $mgml^{-1}$ KTFA was prepared in THF. A solution of the PVAc sample in THF at 1 $mgml^{-1}$ was prepared. PVAc solution was mixed with KTFA solution 1:5, before spotting 0.25 µl of this mixture onto a pre-dried 0.25 µl DCTB spot.

For PVA samples, a 'TA30' solution was prepared by mixing 7 parts TFA solution (0.01% trifluoracetic acid in water) with 3 parts acetonitrile. PVA samples were dissolved in TA30 at concentration 2 mg/ml. A saturated solution of 2,5-DHB in TA30 was prepared. A 2 mg/ml solution of KTFA in TA30 was prepared. The sample, salt and matrix solutions were mixed at ratio 1:1:10 respectively and 1 µl of this solution was spotted onto the plate. The plate was dried under an extractor hood at room temperature. KTFA was used as a cationization agent because this reliably produced a single distribution rather than various H+, Na+, and K+ adducts.

For PVA-protein conjugates, saturated solutions of CHCA and super-DHB were prepared in acetonitrile, mixed 50:50 v:v, and 0.2 µl of this solution was spotted on the plate. Saturated solutions of CHCA and super-DHB were prepared in 2.5% TFA aqueous solution. Then, 0.5 µl CHCA/TFA, 0.5 µl sDHB/TFA, 0.5 µl 10 $mgml^{-1}$ ammonium citrate dibasic, and 0.5 µl of conjugate solution were spotted on top of the dried matrix crystals and dried under an extractor hood.

Biolayer Interferometry

Biolayer Interferometry experiments were performed using an Octet® Red 96 instrument and standard streptavidin (SA) Biosensors. Biotin, PVA and Biotin-PVA samples were dissolved in 1x phosphate buffer saline. PVA and biotin-PVA solutions were made at 25 μgml^{1} (~15 μM) A solution of biotin was prepared at 5 μgml^{-1} (~20 μM). Streptavidin sensors were exposed first to PBS for 10 minutes for a baseline, then to the analyte for 5 minutes, then back to PBS for 10 minutes for dissociation.

SDS-PAGE

SDS-PAGE was performed using a Bio-Rad Mini-PROTEIN precast gel, with MES-SDS running buffer. The gel was stained with NuPAGE LDS sample buffer with 5% 2-mercaptoethanol. The ImageJ gel tool was used to plot the intensity of each lane from a monochrome image of the gel. Pixel positions of proteins from Thermo PageRuler Plus calibrant were used to produce a calibration plot, and calculate molecular weights for BSA-N_3, BSA and BSA-PVA.

BSA-N_3 PVA conjugation and FPLC

BSA-PVA was purified by size-exclusion chromatography using an ÄKTA pure™ protein purification system. The column used was a HiLoad 16/600 Superdex 75 pg equilibrated with 50 mM phosphate 150 mM NaCl pH 7.2 buffer at 4 °C. The experiment was calibrated between 6.5 kDa and 75 kDa using aprotinin, RNAse A, carbonic anhydrase and conalbumin. The injection volume was 0.5 ml.

250 μl DBCO-PVA, 1 mgml^{-1} in running buffer, was mixed with 250 μl BSA-N_3, 0.25 mgml^{-1} in running buffer. The solution was incubated at 4 °C overnight before running. Unmodified BSA, BSA-N_3 and DBCO-PVA alone were also run for comparison. For BSA-PVA, the protein fractions were collected, desalted using a Cytiva PD-10 Sephadex G-25 column, lyophilized, and used for splat assay.

'Splat' IRI assay

After redissolving the lyophilized BSA-PVA fraction from FPLC in PBS, the approximate protein concentration was measured to be 0.7 mgml^{-1} using a Thermo Scientific Nanodrop ND2000 spectrophotometer (280 nm). The BSA control solution was made to be the same concentration.

A 20-50 μm thin ice wafer is produced on a precooled −78 °C glass coverslip by 'splatting' a 10 μL sample solution in phosphate-buffered saline (PBS) from a height of 1 m. This thin ice on coverslip is transferred to a cryostage and annealed at −8 °C for 30 minutes. The process was monitored by a 20x microscope objective attached with a camera and a photo was taken at the start and the end of the experiment. Mean grain area was measured using Cellpose, a deep learning-based segmentation program, in addition to Fiji and LabelstoROIs (Fiji plugin).[1–3] The relative size of ice crystal in the sample was compared to that of PBS.

RAFT/MADIX agent synthesis

Synthesis of ECTTMPA

16.13 g (100 mmol) potassium o-ethyl xanthogenate and 7.85 g (47 mmol) 2-bromo-2-methyl propionic acid were dissolved in 200 ml THF and stirred. After a few minutes the yellow solution became cloudy with white precipitate. After 24 hours stirring, the solvent was removed from the slurry using a rotary evaporator at 50 °C and 400 mbar, leaving a yellow mixture. This residue was dissolved in 200 ml water and acidified with 25 ml 1 M H_2SO_4, then extracted with 200 ml ethyl acetate. The yellow ethyl acetate layer was washed with 100 ml slightly acidified (2 ml 1 M H_2SO_4) water followed by 100 ml brine. The solution was dried with magnesium sulfate and the solvent removed under vacuum. The product was recrystallized in 100 ml hexane at 60 °C, then washed twice with hexane at 0 °C and dried under vacuum. 5.19 g (25 mmol) beige crystals were recovered, 53 % yield. NMR was run in $CDCl_3$ (figures S1 and S2). ECTTMPA ^1H NMR ($CDCl_3$, 400 MHz): δ 11.99 (s, 1H), δ 4.60 (q, J = 7.2 Hz, 2H), 1.64 (s, 6H), 1.39 (d, J = 7.2 Hz, 3H). ^{13}C NMR ($CDCl_3$, 400 MHz,): δ 210.32 ($\underline{\mathbf{C}}$=S), δ 180.17 (C=O), δ 69.97 ($\underline{\mathbf{C}}H_2$), δ 53.96 ($\underline{\mathbf{C}}H(CH_3)_2$), δ 25.58 ($CH(\underline{\mathbf{C}}H_3)_2$), δ 13.21 ($CH_2\underline{\mathbf{C}}H_3$). ESI-MS ion in negative mode –M/Z = 207.0 (figure S3).

Synthesis of PFP-ECTTMPA

2.01 g ECTTMPA (9.6 mmol), 61.4 mg DMAP (0.5 mmol), 2.26 g (12.2 mmol) pentafluorophenol and 2.36 g (12.3 mmol) EDC were dissolved in 40 ml DCM. The solution was degassed for 20 minutes and left stirring at 500 rpm at room temperature (21 °C) in darkness for 48 hours. The solid precipitate was filtered out, then the DCM was removed from the filtrate under vacuum. The residue was redissolved in 60 ml diethyl ether, and washed with 60 ml saturated sodium hydrogen carbonate, followed by 3 washes of 50 ml water, and finally 50 ml brine. The organic phase was dried with $MgSO_4$ then the solvent removed under vacuum. The impure product was recrystallized by dissolving in hot ethanol, cooling to 0 °C and adding

water dropwise until the product began to crystallize, after which it was left to crystallize for 2 days then isolated by Buchner filtration. 1.92 g (5.2 mmol) white crystals were recovered, 21 % yield. NMR was run in $CDCl_3$ (figures S4, S5 and S6). PFP-ECTTMPA ^{1}H NMR ($CDCl_3$, 400 MHz): δ 4.64 (q, J = 7.1 Hz, 2H), δ 1.78 (s, 1H), δ 1.38 (t, J = 7.1 Hz, 3H). ^{13}C NMR ($CDCl_3$, 400 MHz): δ 209.23 (C=S), δ 169.86 (C=O), δ 70.46 (CH_2), δ 53.62 ($\underline{\mathbf{C}}(CH_3)_2$), δ 25.74 ($C(\underline{\mathbf{C}}H_3)_2$), δ 13.04 ($CH_3$). ^{19}F NMR ($CDCl_3$, 400 MHz): δ 153.3 (m, 2F), δ 167.4 (t, 1F), δ (m, 2F). ESI-MS ion in positive mode – M/Z = 397.1 (Na^+ ion) (figure S7).

PET-RAFT of vinyl acetate

Photopolymerization reactions were performed in a HepatoChem PhotoRedOx box using a 450 nm LED lamp, model HCK1012-01-002 P201-18-2, 18W, with specified relative irradiance 34 mW/cm². In reaction vessels with 6 cm² surface area, absolute irradiance is approximately 200 mW.

$PVAc_{19}$-Xan

20.02 g (0.232 mol 25 eq.) vinyl acetate, 40.37 g DMSO, 1.93 g (9.2 mmol, 1 eq.) ECTTMPA and 0.22 g (0.47 mmol, 0.05 eq.) bismuth oxide were added to a 60 ml glass reaction vessel with a magnetic stirrer bar and mixed vigorously until the bismuth oxide was well suspended. The vessel was fitted with a rubber septum and the reaction was stirred and exposed to 450 nm light for 18 hours. A sample as taken for conversion NMR, then the catalyst was removed by centrifugation at 10,000 rpm. The polymer was precipitated from the DMSO in water, redissolved in THF and precipitated in water again, then redissolved and precipitated again 3 times in THF / hexane. The polymer was then dried under vacuum, yielding 15.3 g PVAc.

PFP-$PVAc_{23}$-Xan

3.00 g (34.8 mmol, 25 eq.) vinyl acetate, 0.525 g (1.40 mmol, 1 eq.) PFP-ECTTMPA, 3.90 g DMSO and 12.8 mg (465.96 $gmol^{-1}$, 0.027 mmol, 0.019 eq.) bismuth oxide was mixed in an 8 ml vial. A magnetic stirrer bar was added, and the vial was fitted with a rubber septum. The vial was exposed to 450-455 nm light with moderate stirring. After 16 hours, a sample was taken for determining conversion by ^{1}H NMR, then the polymer solution was centrifuged at 10,000 rpm for 5 minutes to remove the suspended bismuth oxide. In addition to yellow bismuth oxide, some less dense black particulate was also removed from the solution. The polymer was precipitated from the supernatant in 25 ml water to remove the DMSO, and centrifuged again. The polymer was precipitated once more using THF / water then twice with THF / pentane. The polymer was dried under vacuum, yielding 2.73 g PVAc.

Photoinduced xanthate removal from PVAc

Removal of xanthates was performed in a Heptatochem photoredox box using a 18W 380 nm LED lamp, model HCK1012-01-013 with specified relative irradiance of 8 $mWcm^{-2}$. 8 ml glass vials with a rubber septum were used, which when filled have approximately 6 cm^2 surface area, with an absolute irradiance per vial of 50 mW.

$PVAc_{19}$-H

126 mg DS186 (approx. 1850 $gmol^{-1}$, 0.068 mmol, 1 eq.) was dissolved in 1ml methanol, and added to a vial containing 385 mg (2.15 mmol, 31.6 eq.) 1-ethylpiperidine hypophosphite (EPHP). The solution was degassed by nitrogen sparging for 10 minutes, then stirred and exposed to 380 nm light for 3 hours. The polymer was precipitated in water, then redissolved in methanol and precipitated in water again. After shortly drying under vacuum, the polymer was redissolved in THF and precipitated in hexane, washed with hexane and dried under vacuum again yielding 90 mg PVAc.

PFP-$PVAc_{23}$-H

1.04 g (approx. 2400 $gmol^{-1}$ 0.43 mmol, 1 eq) PFP-PVAc-Xan was dissolved in 3 ml methanol. 1.34 g (179.20 g/mol 7.47 mmol, 17 eq) EPHP was dissolved in 3 ml methanol. Both solutions were combined in 8 ml vial, a magnetic stir bar was added, and rubber septum fitted, then the solution was degassed by nitrogen sparging for 20 minutes. The solution was then exposed to 380 nm light in the photoreactor with moderate stirring. A sample was used for ^{1}H NMR after 5 hours which confirmed the reaction was complete, and exposure was stopped at 6 hours. The PVAc was precipitated from the reaction mixture in water, rinsed with pentane centrifuged, then precipitated twice in THF / pentane. The product was dried under vacuum for 2 hours, yielding 0.954 g polymer.

Polymer modifications

Synthesis of Bzl-$PVAc_{23}$

54.9 mg PFP-$PVAc_{23}$-H (0.024 mmol) was dissolved in 1 ml dioxane. 13 µl benzylamine (~12.7 mg, 0.12 mmol, 5 eq) was added. The solution was heated to 50 °C for 2 hours with gentle stirring in a sealed vessel. ^{19}F NMR of the reaction mixture showed complete transformation of the PFP-ester to free pentafluorophenol. After 3.5 hours, the polymer was precipitated in 6 ml water, then washed several times with pentane, and dried under vacuum,

leaving 55 mg Bzl-$PVAc_{23}$. 1.5 mg product was dissolved in $CDCl_3$ for NMR analysis. The remaining polymer was immediately deacetylated.

Deacetylation of Bzl-$PVAc_{23}$ to Bzl-PVA_{23}

50 mg Bzl-$PVAc_{23}$ was dissolved in 160 µl methanol, then 100 µl hydrazine hydrate solution was added. The solution was stirred at room temperature for 16 hours. The reaction mixture was precipitated in 1.5 ml isopropyl alcohol, then the solid was washed with 1 ml isopropyl alcohol, washed with 1ml pentane twice, then dried under vacuum.

Synthesis of Biotin-PVA_{23}-H

4.3 mg pentylamine-biotin and 19.7 mg PFP-PVAc-H were dissolved in 500 µl DMF, 500 µl, and heated to 50 °C. After 21 hours, a sample of the reaction mixture was taken for ^{19}F NMR which showed complete hydrolysis of the PFP ester. The PVAc was precipitated in 1.5 ml water and after centrifugation was dried under vacuum. The entire sample was dissolved in 650 µl $CDCl_3$ for NMR experiments, after which the solvent was removed under reduced pressure. The PVAc was redissolved in 100 µl methanol, then 50 µl hydrazine hydrate solution was added. The solution was stirred for 3 hours at 50 °C, then the polymer was precipitated in 1.5 ml isopropyl alcohol, cooled with liquid nitrogen to encourage complete precipitation then centrifuged and dried under vacuum. 3.4 mg biotin-PVA recovered, and analyzed by ^{1}H NMR in D_2O, and MALDI-TOF.

Synthesis of DBCO-PVA_{23}

26.1 mg PFP-$PVAc_{23}$-H dissolved in 1 ml DMF. 4.1 mg DBCO amine was added. The mixture was agitated on a roller until completely dissolved, then heated to 50 °C and stirred for 2 hours 30 minutes. The DMF was removed under vacuum. The product was dissolved in 1 ml methanol, cooled in an ice bath, and 100 µl 5.4 M methanolic sodium methoxide solution was added. The solution was stirred for 10 minutes, then stirred at 25 °C for 1 hour. The product was precipitated in isopropyl alcohol cooled with liquid nitrogen, and centrifuged. The pellet was redissolved in water 1 ml, washed with ethyl acetate 1 ml three times, and lyophilized.

Synthesis of BG-PVA_{23}

176 mg FP-$PVAc_{23}$-H and 32.5 mg BG-NH_2 were dissolved in 6 ml DMF, and stirred at 50 °C for 2 hours, at which point a sample was taken for ^{19}F NMR which showed an incomplete reaction. A further 39 mg BG-NH_2 was added, and the reaction stirred for a further 4 hours, after which ^{19}F NMR showed completion. The DMF was removed at 50 °C under vacuum.

6 ml methanol was added, the solution was cooled to 0 °C and 600 µl 5.4 M methanolic sodium methoxide solution was added. The reaction was stirred for 16 hours. The solution was neutralized by adding 3 ml water and amberlite IR 120 H^+ resin. Then the solution was precipitated in cold acetone and centrifuged. The pellet was washed with methanol and dried under vacuum. 31 mg BG-PVA was recovered.

Deacetylation of unmodified PFP-PVAc$_{23}$-H

50.4 mg PFP-PVAc-H was dissolved in 200 µl methanol, and 100 µl hydrazine hydrate 50-60 % solution was added. The solution was stirred at 50 °C for 4 hours then precipitated in IPA, redissolved in 100 µl water, precipitated in 2 ml IPA and dried under vacuum. 20 mg PVA was recovered.

Synthesis of longer PVAc and modifications

Synthesis of higher molecular weight PFP-PVAc$_{110}$-Xan

91.8 mg (0.278 mmol, 1 eq.) PFP-ECTTMPA, 3.59 g vinyl acetate (150 eq.), 0.3 ml anhydrous DMSO and 26 mg bismuth oxide were mixed in an 8 ml vial. A micro stir bar was added and the vial fitted with a rubber septum. The vial was exposed to blue 450 nm light in a homemade bottom-lit LED photoreactor drawing 1.65w. The temperature reached 40C as measured by thermocouple. After 3 hours, ^{1}H NMR showed 70% conversion and ^{19}F NMR showed no hydrolysis of the PFP ester. SEC was run of the reaction mixture (figure S28). The polymer was precipitated twice from ethyl acetate into pentane, and 2.49 g PVAc was recovered after drying under vacuum.

Removal of xanthate from PFP-PVAc$_{110}$-Xan

243 mg PFP-PVAc$_{110}$-Xan was dissolved in 2 ml THF, then 2 ml IPA and 330 mg EPHP were added. The solution was degassed by sparging with nitrogen for 10 minutes, then exposed to UV 380 nm light in the photoreactor with moderate stirring for 24 hours. The polymer was precipitated in water and washed with pentane before drying under vacuum, 210 mg polymer was recovered.

Synthesis of BG-PVA$_{110}$

28 mg long PFP-PVAc$_{110}$-H was dissolved in 2 ml DMF. 10.5 mg BG-NH_2 was added. The solution was heated to 50 °C and stirred for 20 hours. The polymer was precipitated in water and dried under vacuum. The polymer was redissolved in 1 ml methanol, then 200 µl 5.4 M methanolic sodium methoxide was added. The solution was stirred very vigorously for 24 hours

at room temperature, then precipitated in IPA, washed twice with 5 ml ethanol and dried under vacuum. 8.3 mg BG-PVA was recovered, which was analyzed by ^{1}H NMR and MALDI-TOF MS.

Conjugation of BG-PVA$_{110}$ to snap tag protein

5 µl 1 mgml^{-1} snap tag protein in phosphate buffer saline was mixed with 10 µl 6 mgml^{-1} BG-PVA in phosphate buffer saline and 1 µl 10 mgml^{-1} TCEP in water and incubated at 37 °C for 30 minutes before MALDI-TOF analysis.

Additional Data and Characterization

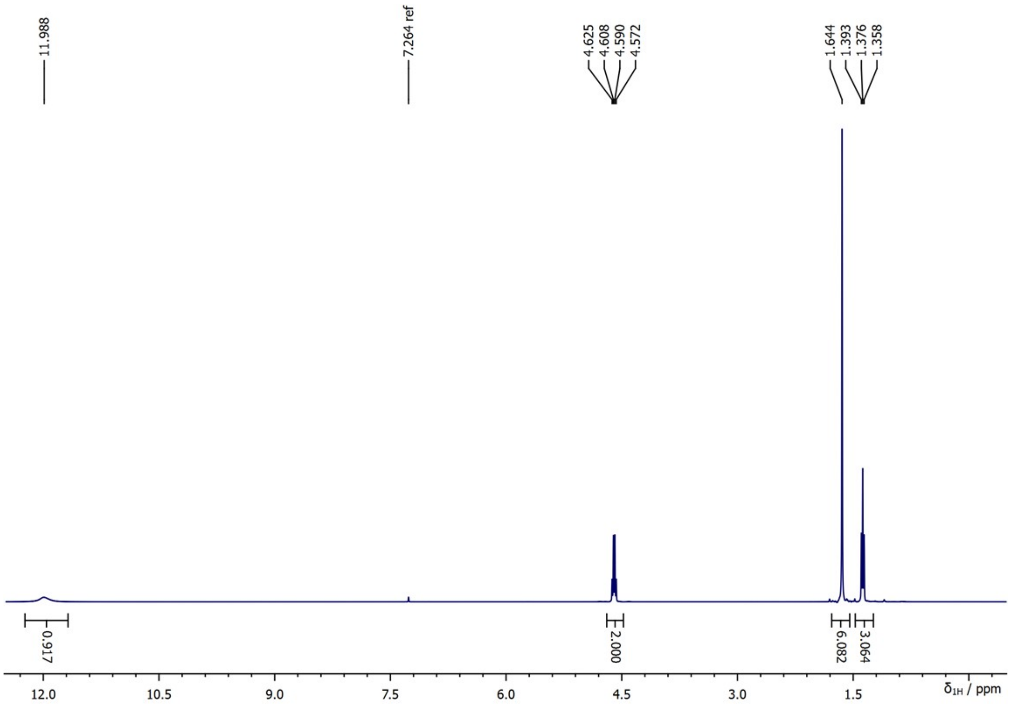

Figure S1. ECTTMPA. ^{1}H NMR ($CDCl_3$, 400 MHz): δ 11.99 (s, 1H), δ 4.60 (q, J = 7.2 Hz, 2H), 1.64 (s, 6H), 1.39 (d, J = 7.2 Hz, 3H).

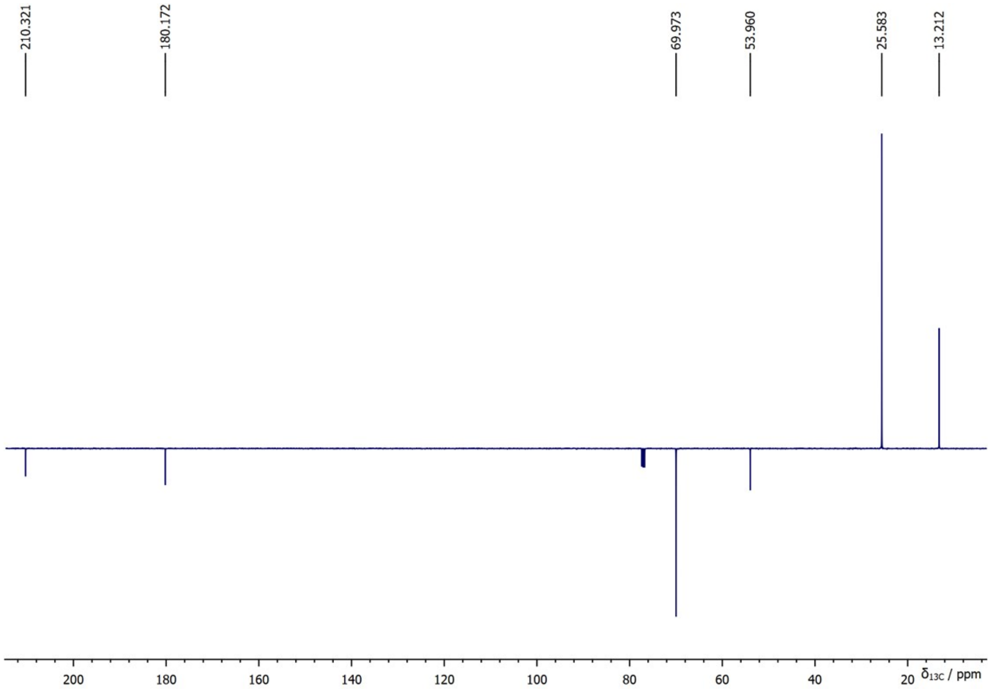

Figure S2. ECTTMPA. ^{13}C NMR ($CDCl_3$, 400 MHz,): δ 210.32 (**C**=S), δ 180.17 (C=O), δ 69.97 ($\mathbf{C}H_2$), δ 53.96 ($\mathbf{C}H(CH_3)_2$), δ 25.58 ($CH(\mathbf{C}H_3)_2$), δ 13.21 ($CH_2\mathbf{C}H_3$).

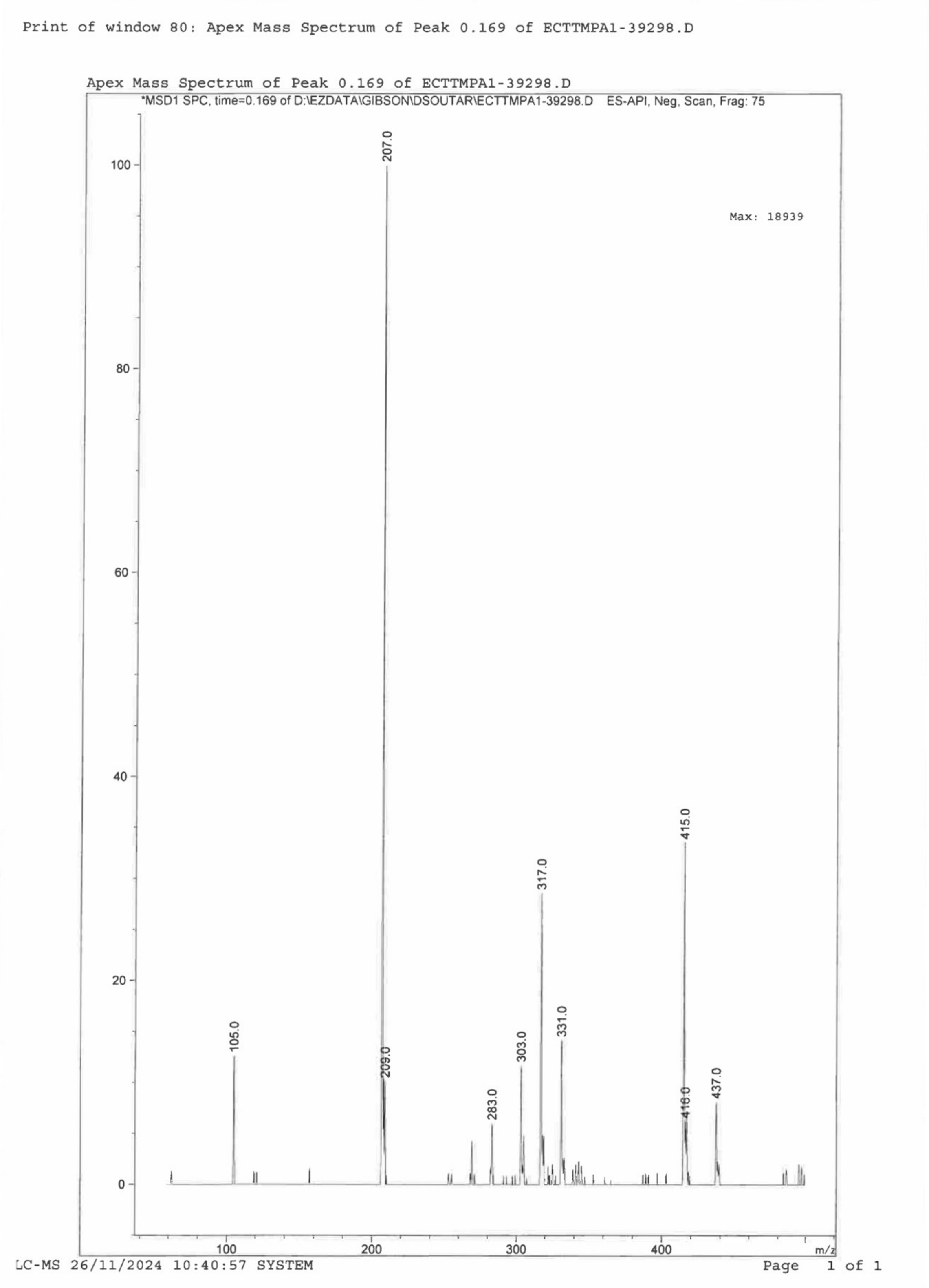

Figure S3. Negative mode ESI-MS mass spectrum of ECTTMPA. $C_7H_{11}O_3S_2^-$ peak = 207.02

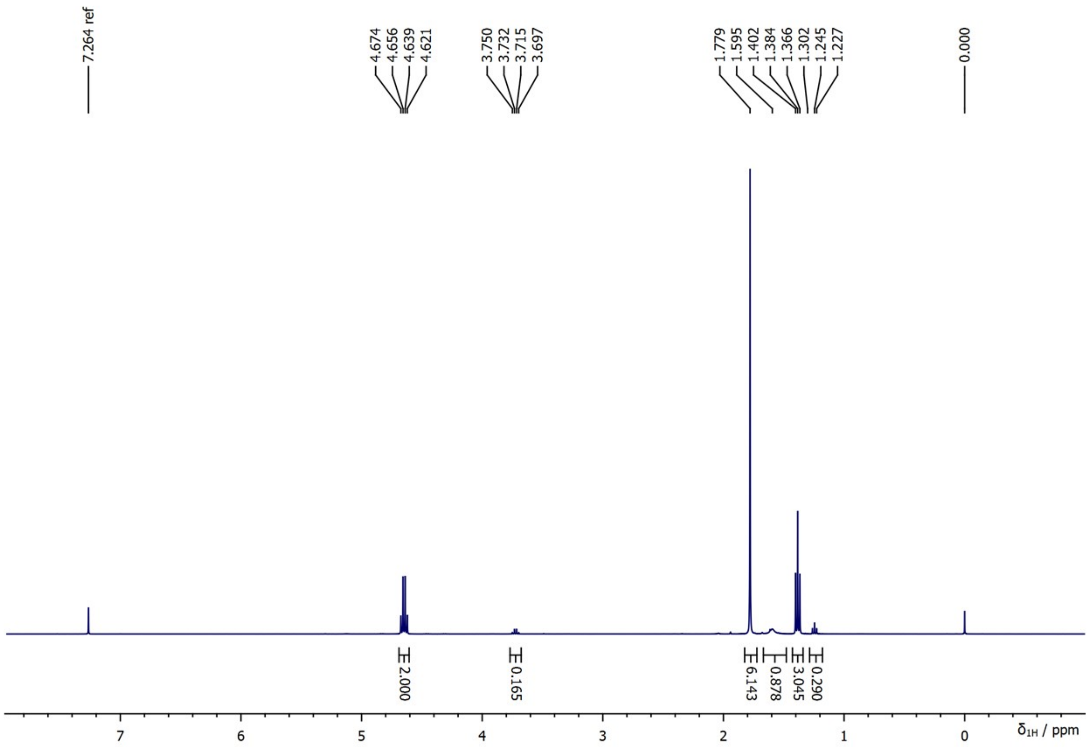

Figure S4. PFP-ECTTMPA. ^{1}H NMR ($CDCl_3$, 400 MHz): δ 4.64 (q, J = 7.1 Hz, 2H), δ 1.78 (s, 1H), δ 1.38 (t, J = 7.1 Hz, 3H)

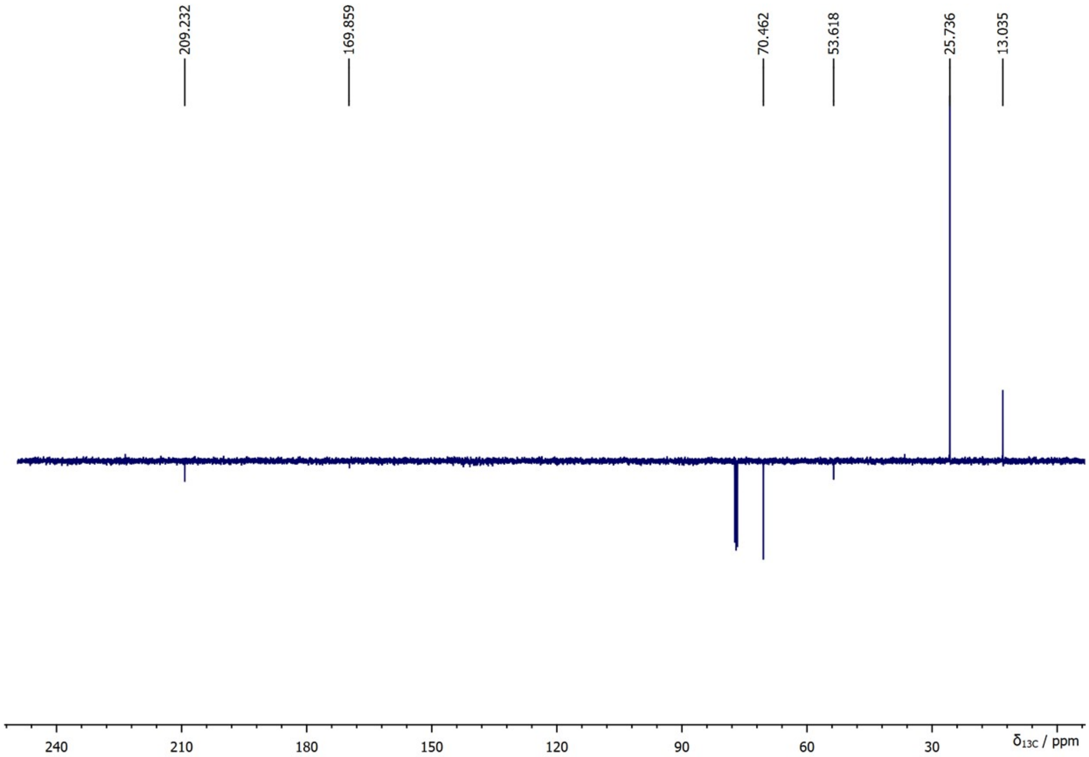

Figure S5. PFP-ECTTMPA. ^{13}C NMR ($CDCl_3$, 400 MHz): δ 209.23 (C=S), δ 169.86 (C=O), δ 70.46 (CH_2), δ 53.62 ($\underline{\mathbf{C}}(CH_3)_2$), δ 25.74 ($C(\underline{\mathbf{C}}H_3)_2$), δ 13.04 ($CH_3$).

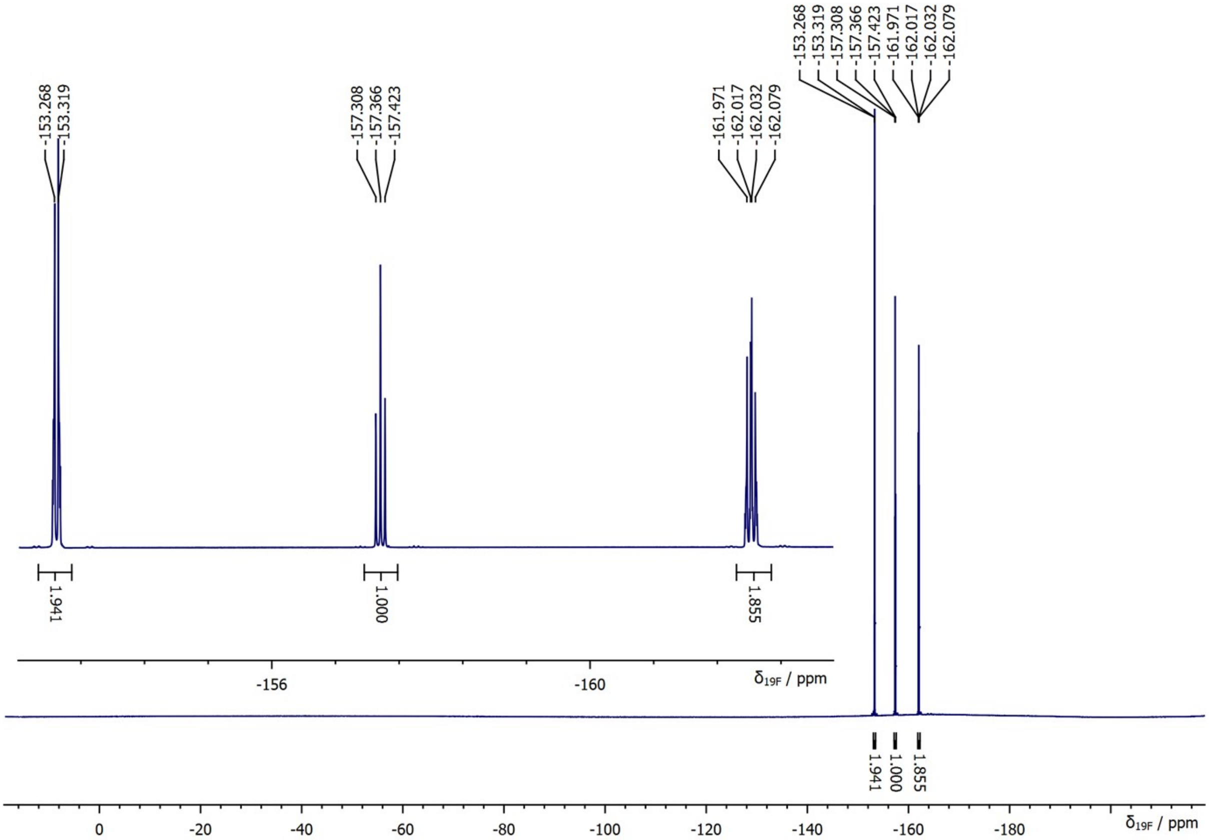

Figure S6. PFP-ECTTMPA. ^{19}F NMR ($CDCl_3$, 400 MHz): δ 153.3 (m, 2F), δ 167.4 (t, 1F), δ (m, 2F)

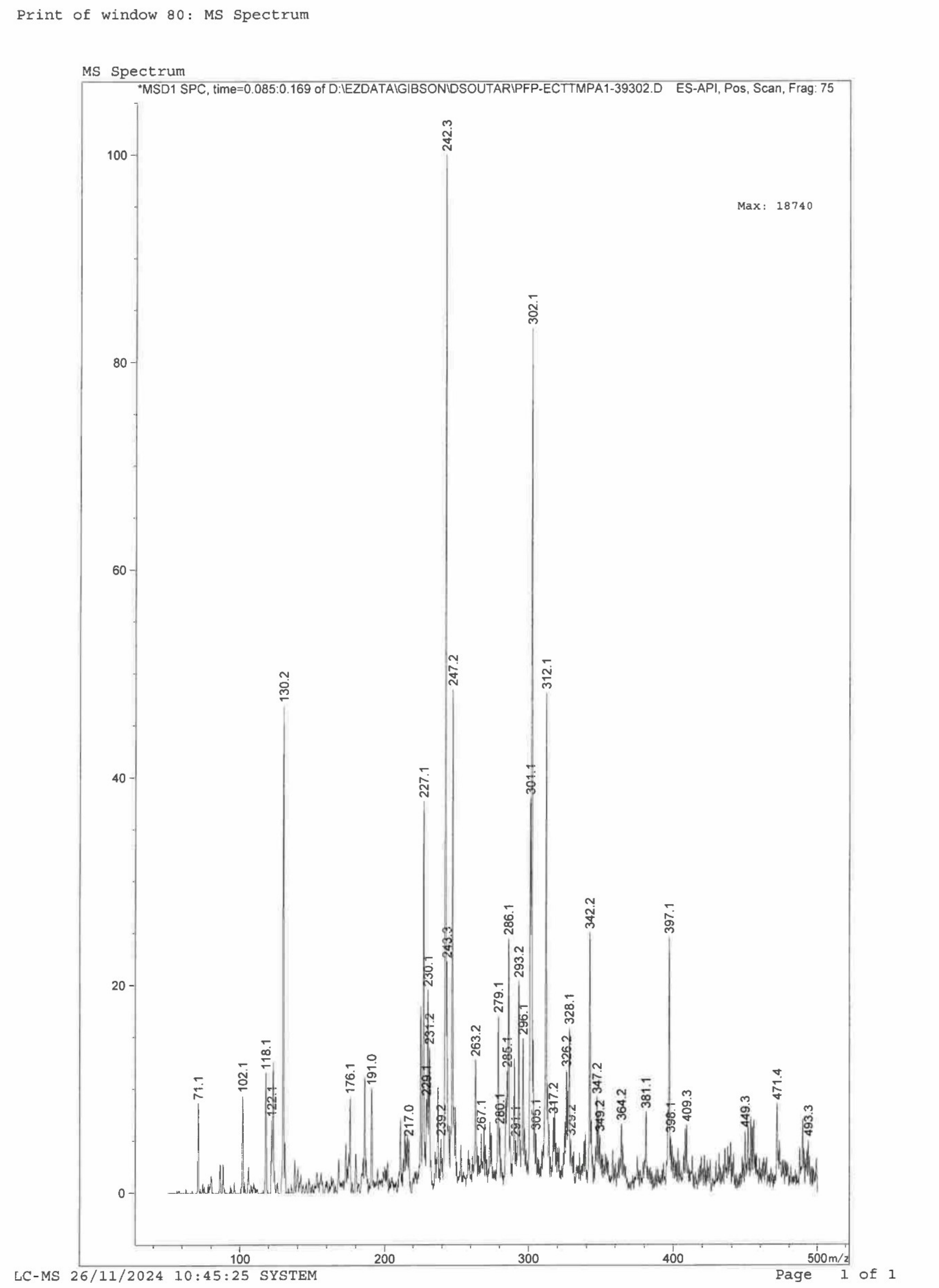

Figure S7. Positive mode ESI-MS mass spectrum of PFP-ECTTMPA. $C_{13}H_{11}F_5O_3S_2$ Na^+ ion = 397.00

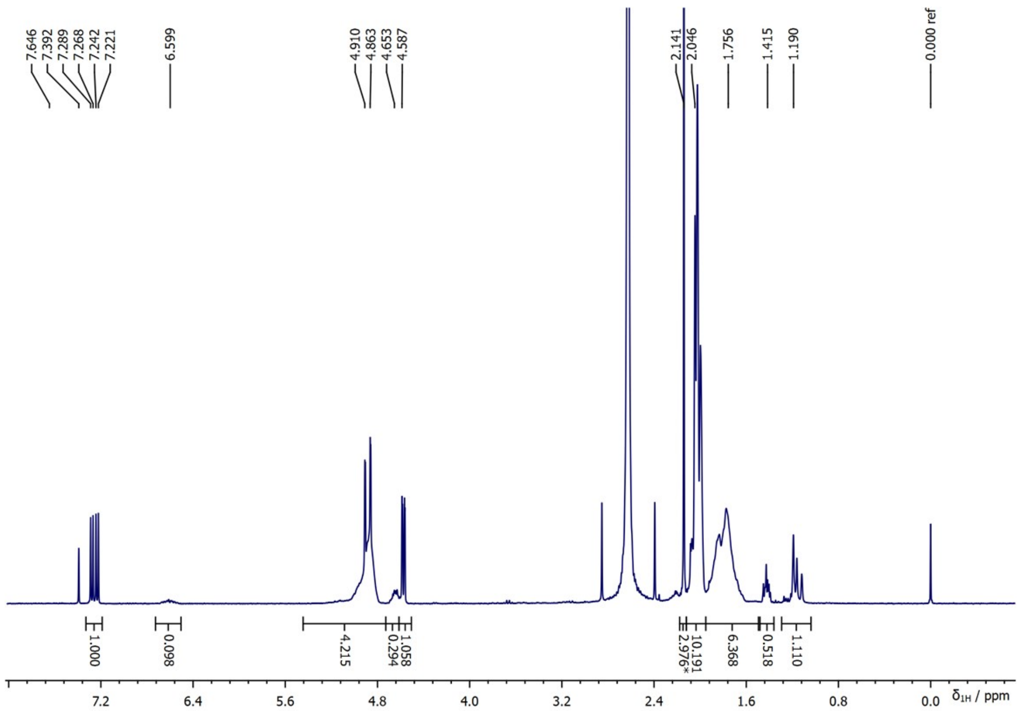

Figure S8. ^{1}H NMR spectrum of ECTTMPA PVAc reaction mixture after polymerization. Used to calculate conversion. Based on VAc alkene : PVAc CH, 76%. Based on VAc CH_3 : PVAc CH_3, 77%

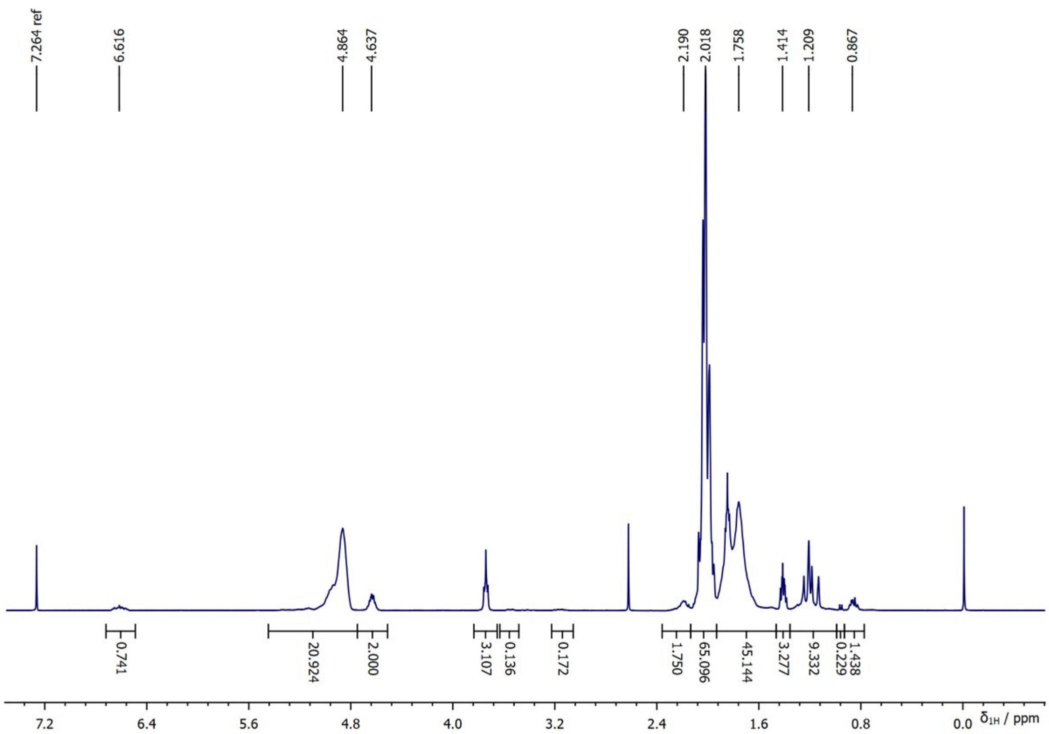

Figure S9. Precipitated polymer $PVAc_{19}$-Xan. ^{1}H NMR ($CDCl_3$, 400 MHz) δ 6.61 (1H, C**H**(OAc)-Xan), δ 4.73 - 5.29 (21H, backbone C**H**), δ 4.63 (2H, xanthate OC$\mathbf{H}_2$), δ 2.11 - 2.28 (integral, 1H), δ 1.90 - 2.11 (OAc C$\mathbf{H}_3$, 63H), δ 1.45 - 1.90 (42H, backbone C$\mathbf{H}_2$), δ 1.41 (3H, xanthate $OCH_2C\mathbf{H}_3$), δ 1.20 (6H R group $(C\mathbf{H}_3)_2$).

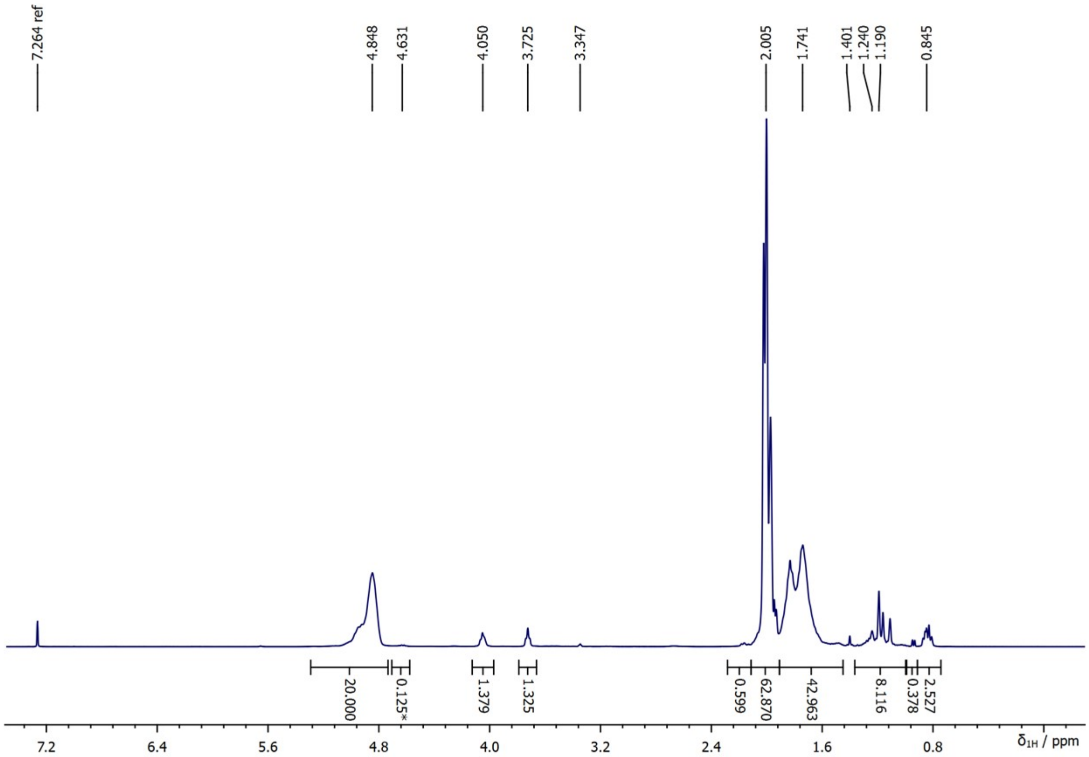

Figure S10. PVAc$_{19}$-H, product of PVAc treated with EPHP and 380 nm ^{1}H NMR (400 MHz, $CDCl_3$) δ 4.85 (20H), δ 4.05 (2H, omega C(OAc)$\mathbf{\underline{H}}_2$) δ 2.00 (63H, OAc C$\mathbf{\underline{H}}_3$) δ 1.74 (backbone C$\mathbf{H}_2$) δ 1.4-1.2 (6H R group (C$\mathbf{H}_3$)$_2$)

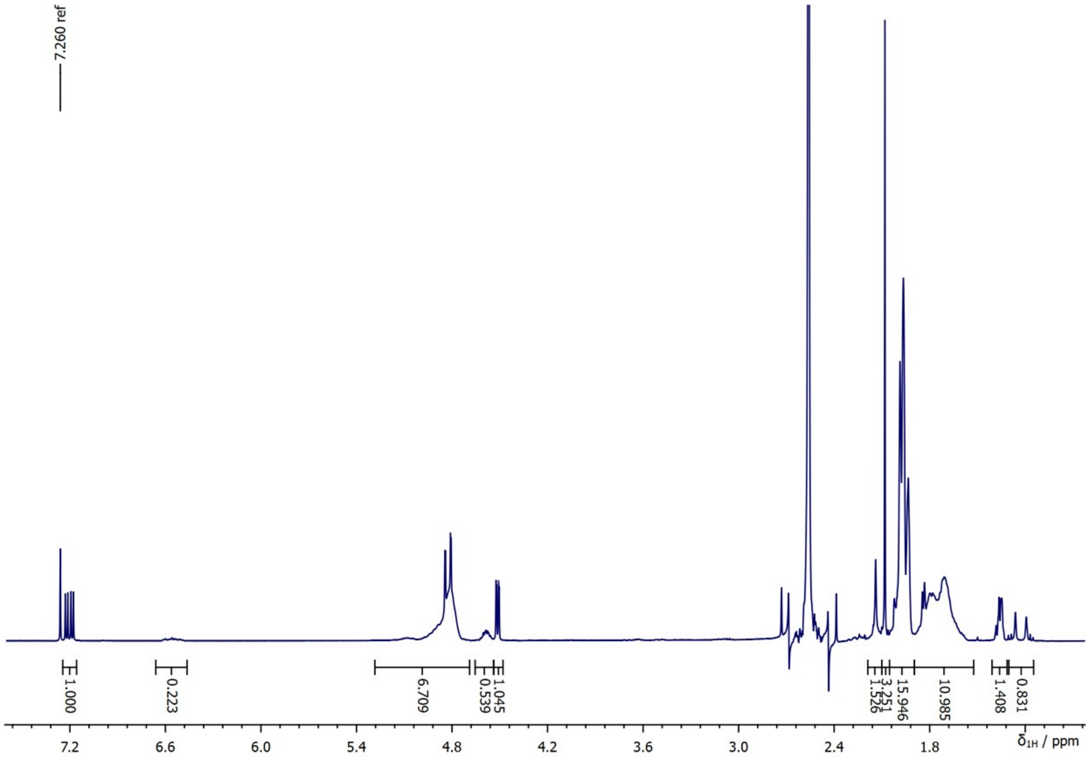

Figure S11. ^{1}H NMR spectrum of PFP-ECTTMPA PVAc reaction mixture after polymerization. Used to calculate conversion. Based on VAc alkene : PVAc CH, 85%. Based on VAc CH_3 : PVAc CH_3, 83%

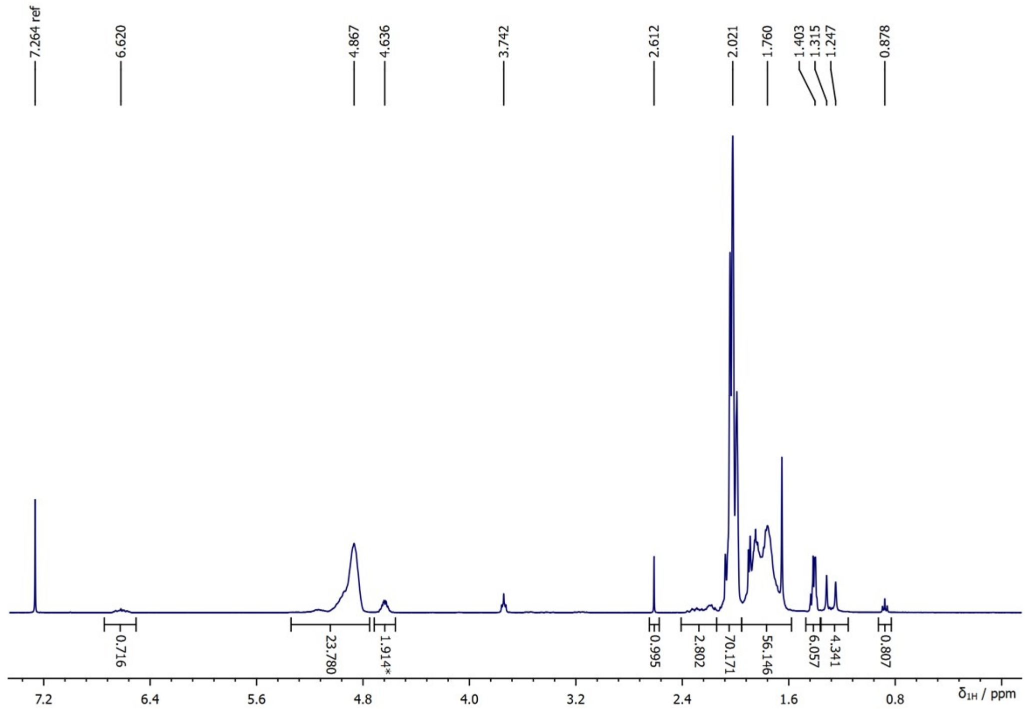

Figure S12. ^{1}H NMR of PFP-PVAc-Xan following precipitation.^{1}H NMR (400 MHz, $CDCl_3$) δ (1H, C(OAc)**H**-Xan), δ 4.87 (23H, backbone C(OAc)**H**), δ 4.64 (2H, xanthate C**H**$_2$), δ 2.02 (70H, OAc C**H**$_3$), δ 1.76 (56H, backbone C**H**$_2$), δ 1.4 - 1.2 (9H, xanthate CH_3 & R group C(C**H**$_3$)$_2$)

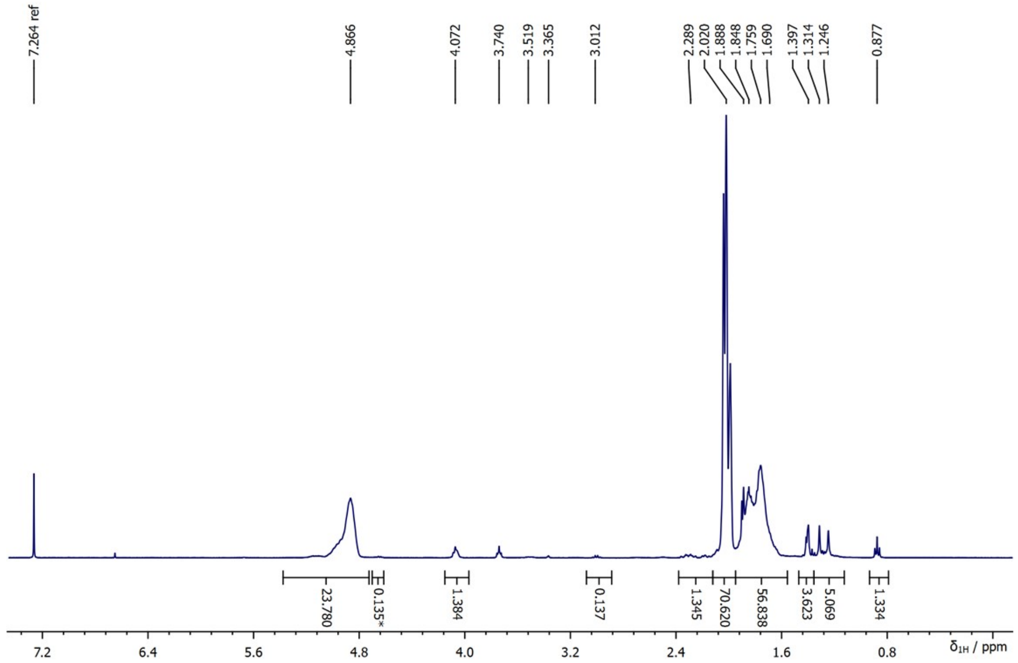

Figure S13. ^{1}H NMR of PFP-PVAc-H. ^{1}H NMR (400 MHz, $CDCl_3$) δ 4.86 (23H, backbone C(OAc)**H**), δ 4.07 (2H, omega C(OAc)**H**$_2$), δ 2.02 (70H, OAc C**H**$_3$), δ 1.76 (56H, backbone C**H**$_2$), δ 1.4 - 1.2 (6H, R group C(C**H**$_3$)$_2$)

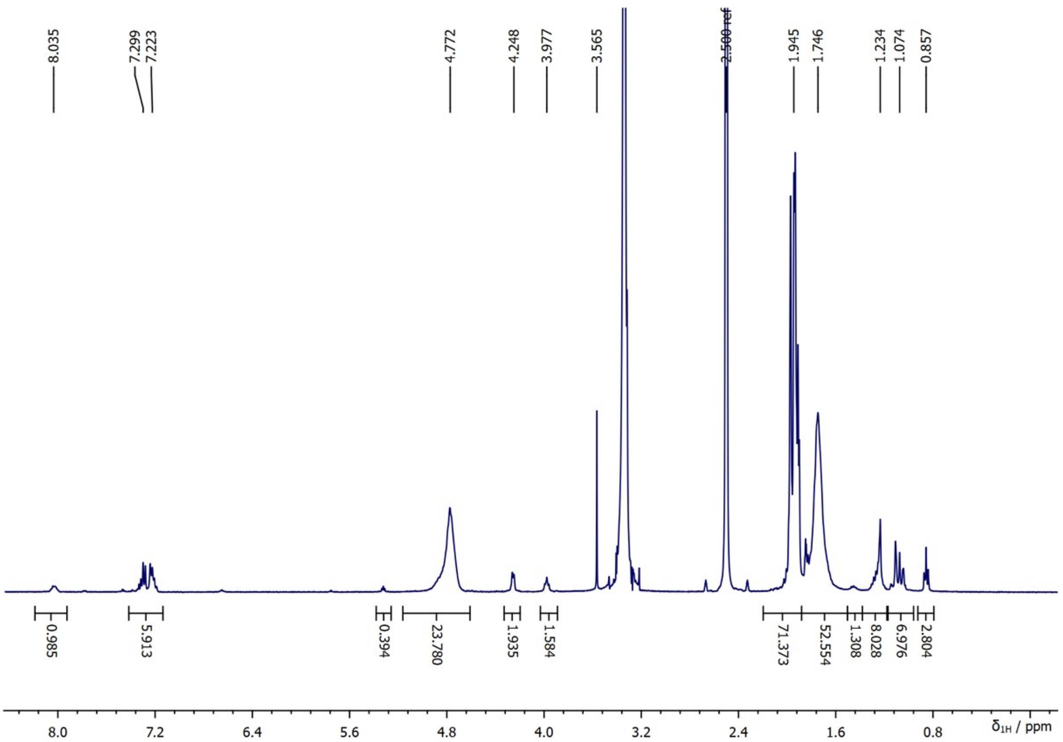

Figure S14. Benzyl-PVAc. ^{1}H NMR (400 MHz, $CDCl_3$) δ 8.04 (1H, N**H**), δ 7.3 (5H, benzyl C**H**), δ 4.77 (24H, backbone C**H**(OAc)), δ 4.25 (2H, benzyl CH_2), δ 3.98 (2H, ω - terminus C(OAc)$\mathbf{H}_2$), δ 1.95 (71H, OAc C$\mathbf{H}_3$), δ 1.75 (52H, backbone C$\mathbf{H}_2$) δ 1.25-1.0 (6H, R group $C(CH_3)_2$)

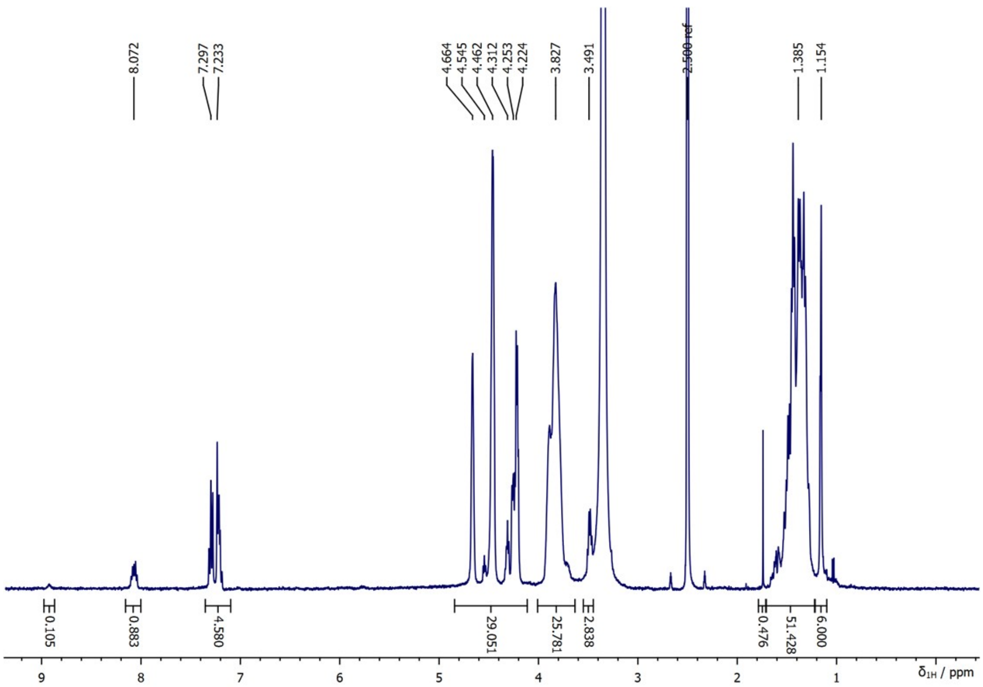

Figure S15. ^{1}H NMR of Benzyl-PVA. ^{1}H NMR (400 MHz, C_2D_6OS): δ 8.07 (1H, NH) , δ 7.25 (m, 5H, benzyl CH), δ 4.6 - 4.2 (CO**H**, mm, mr, rr), δ 3.83 (26H, backbone CH), δ 3.49 (2H, benzyl CH_2), δ 1.39 (52H, backbone CH_2), 1.15 (6H, R group $C(CH_3)_2$)

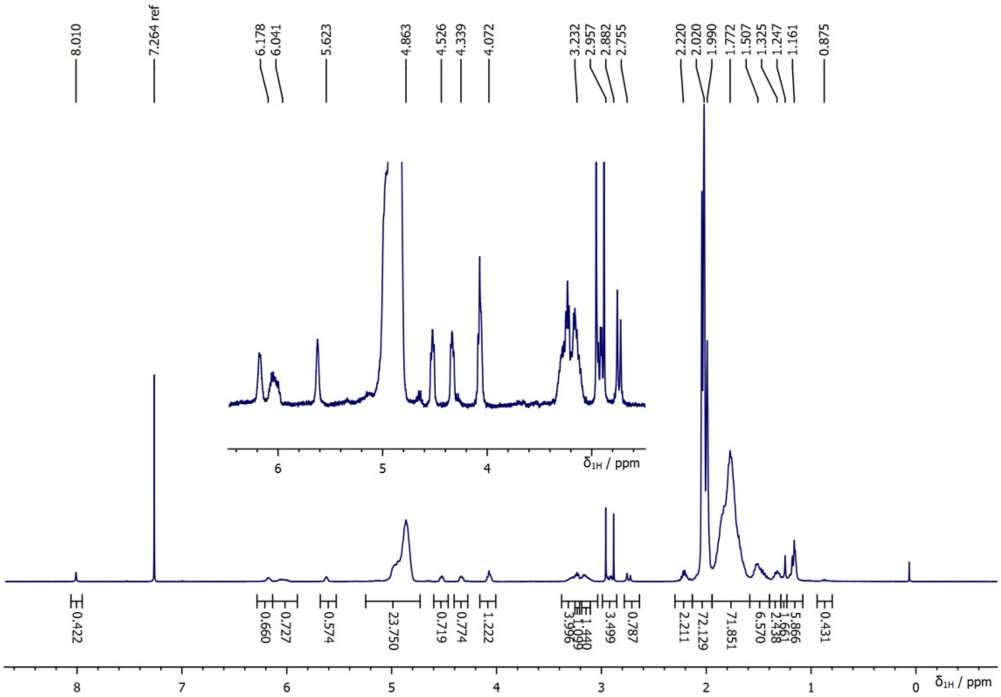

Figure S16. ^{1}H NMR of biotin-modified PVAc. ^{1}H NMR (400 MHz, $CDCl_3$) δ 6.1-5.6 (1H, amide NH), δ 4.86 (backbone CH), δ 4.53 (biotin CH), δ 4.34 (biotin CH), δ 4.07 (biotin CH_2), δ 3.23 (biotin, pentyl), δ 2.96 (biotin) δ 2.76 (biotin), δ 2.22 (biotin) δ 2.02 (OAc CH_3), δ 1.77 (backbone CH_2) 1.50-1.1 (R group $(CH_3)_2$)

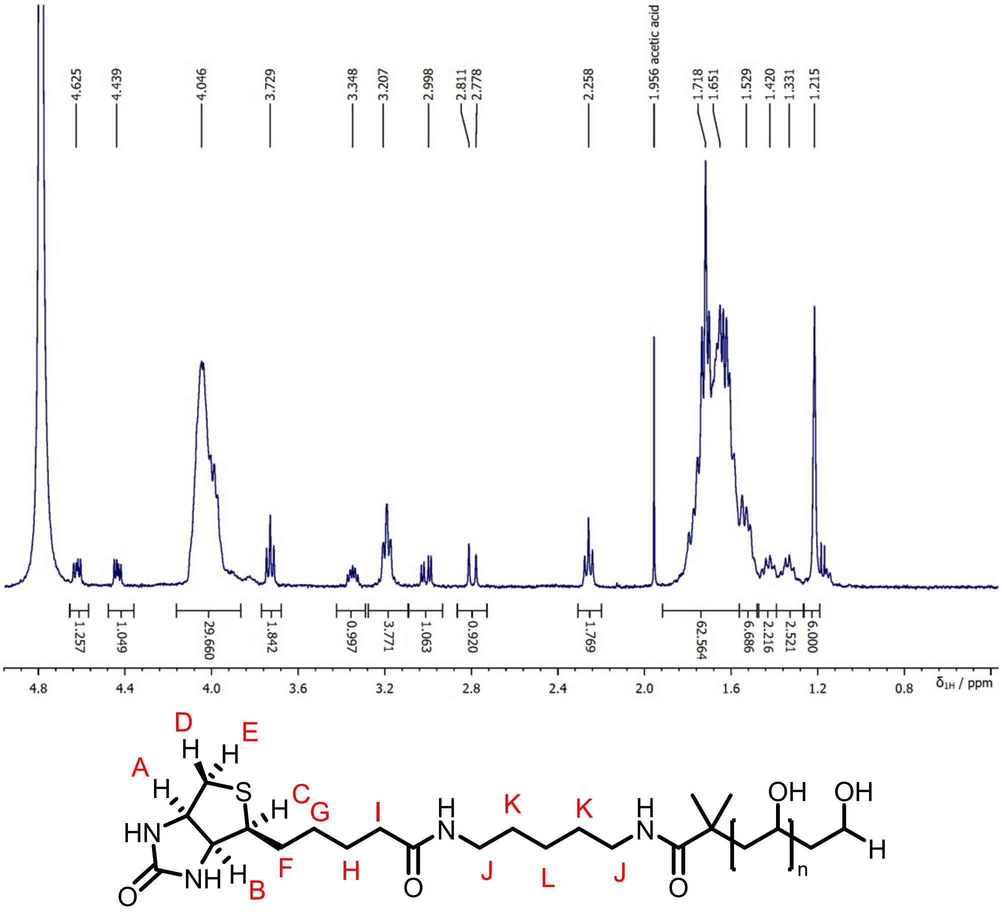

Figure S17. Biotin-modified PVA. ^{1}H NMR (D_2O, 400 MHz) δ 4.63 (dd, 7.9Hz, 4.8Hz, 1H, A), δ 4.44 (dd, 7.8Hz, 4.5Hz, 1H, B), 4.05 (23H backbone C**H**OH), δ 3.73 (t, 6.72 Hz), δ 3.35 (p, 4.4 Hz, 1H, C), δ 3.19 (m, 4H, J), δ 3.00 (dd, 13.1 Hz, 4.9 Hz, D), δ 2.8 (d, 13.1 Hz, E), δ 2.26 (t, 7.2 Hz, 2H, I), δ 1.9 - 1.6 (backbone CH_2), 1.53 (m, 4H, K) δ 1.42 (H), δ 1.33 (L), δ 1.22 (s, R group $C(CH_3)_2$)

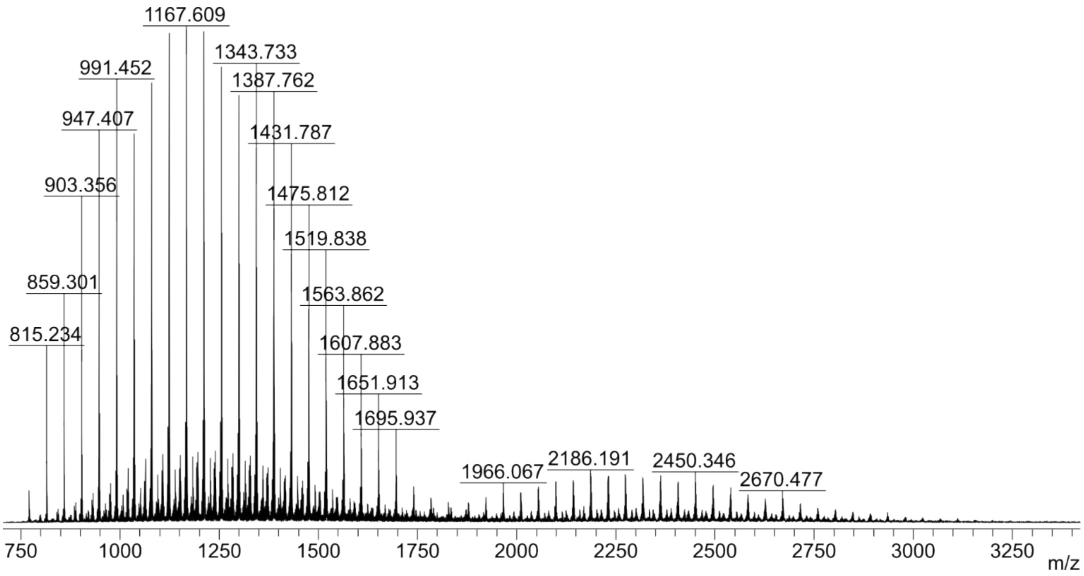

Figure S18. MALDI-TOF of unmodified PVAc-H hydrolysed to PVA by use of hydrazine hydrate.

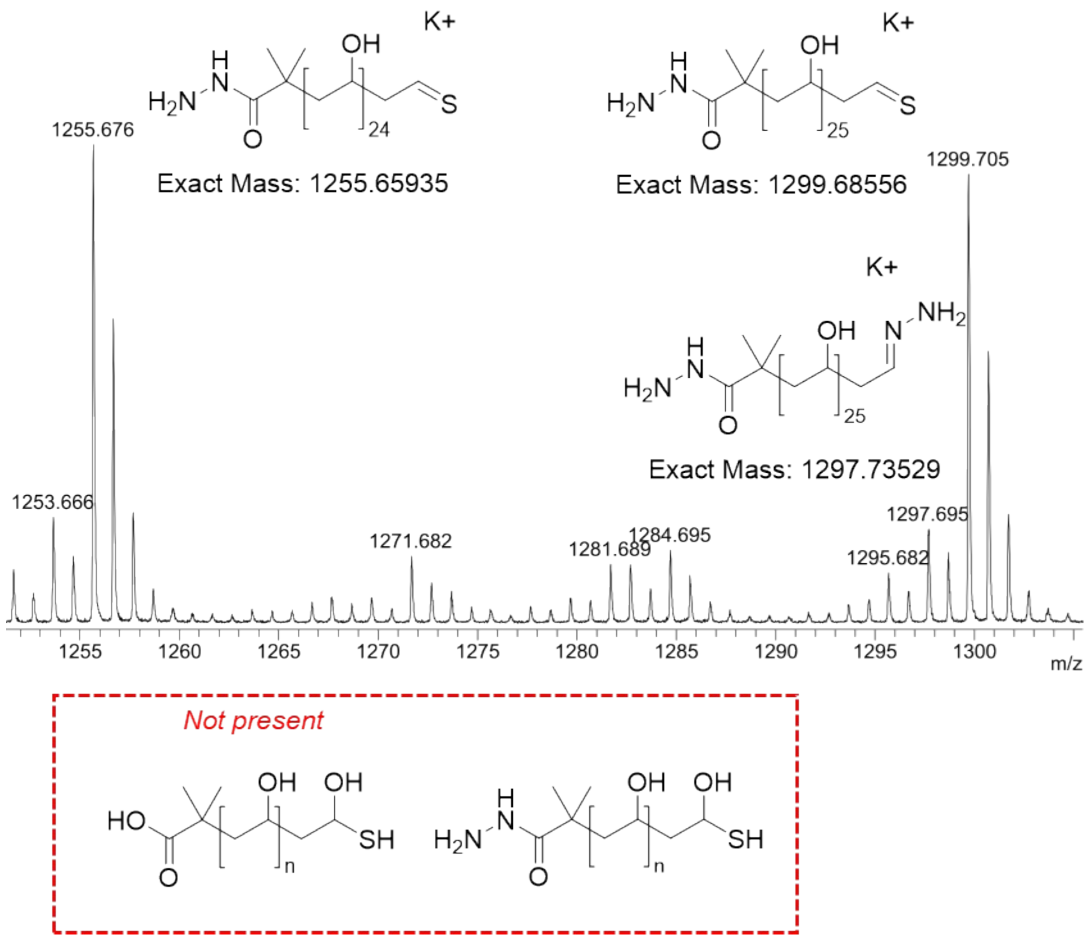

Figure S19. MALDI-TOF of unmodified PVAc-H hydrolysed to PVA by use of hydrazine hydrate showing highest peaks from main distribution. We did not see peaks corresponding to chains with a thiol and alcohol at the chain end.

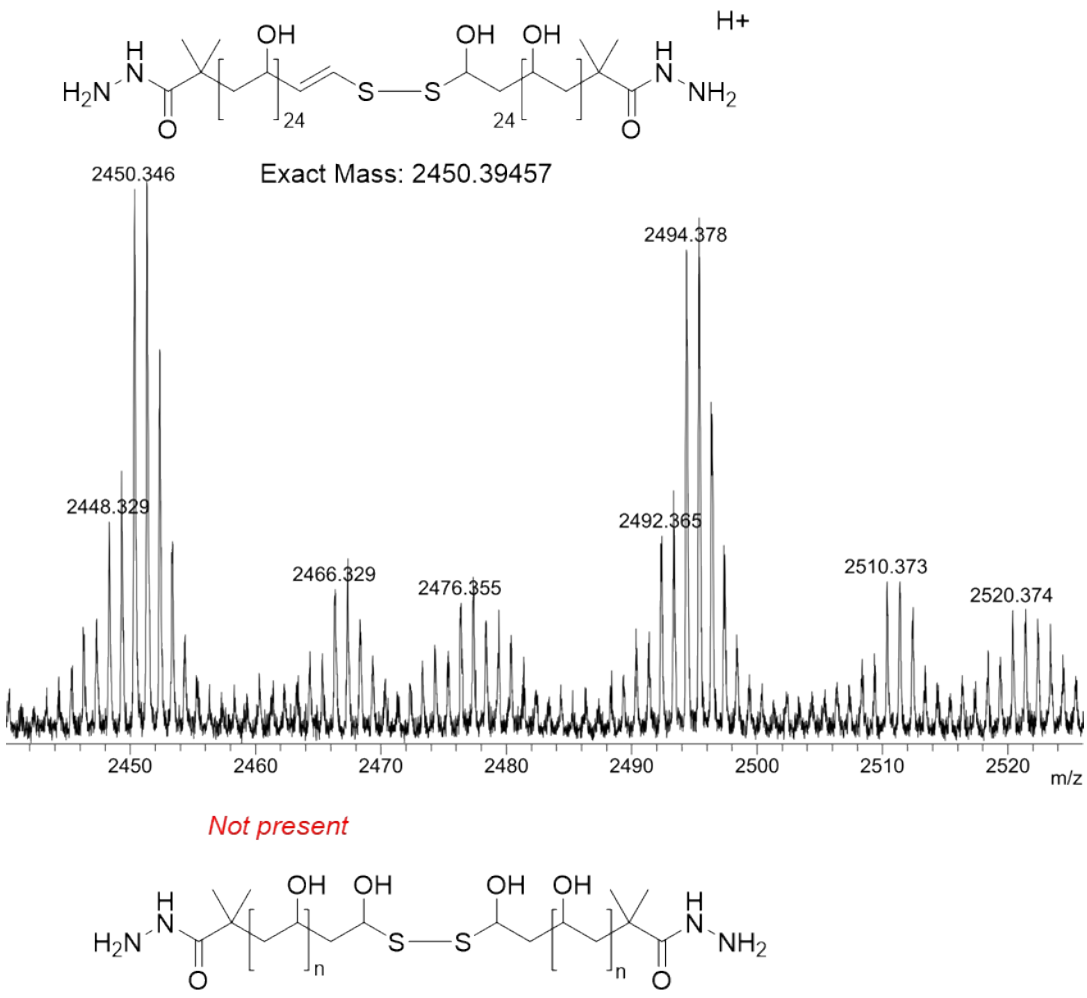

Figure S20. MALDI-TOF of unmodified PVAc-H hydrolysed to PVA by use of hydrazine hydrate showing highest peaks from the second distribution (likely disulfides or product of Michael addition).

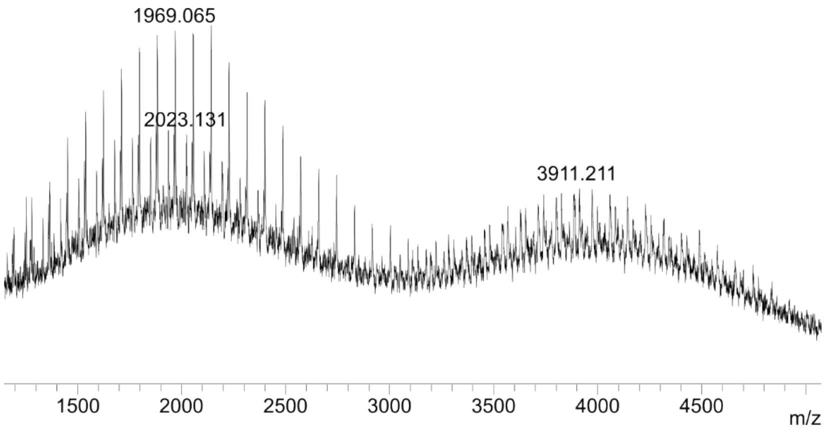

Figure S21. MALDI-TOF of PVAc modified by treatment of hexylamine and N-benzyl acrylamide, without treatment by UV light and EPHP.

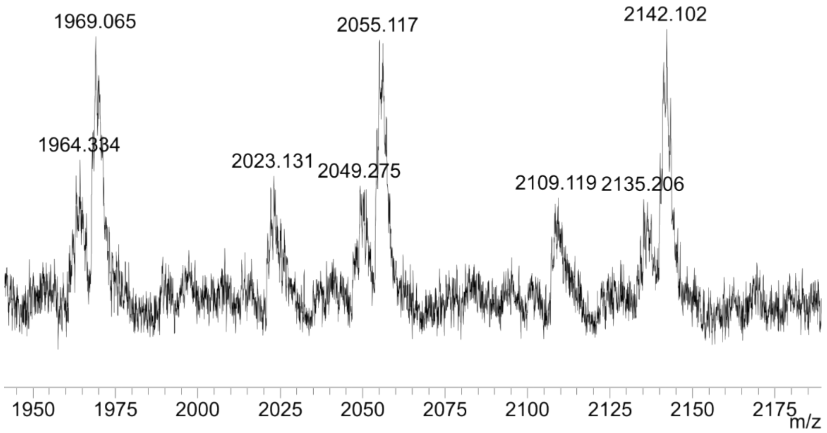

Figure S22. MALDI-TOF of PVAc modified by treatment of hexylamine and *N*-benzyl acrylamide. Peaks from centre of first distribution. Peaks do not correspond to expected masses for a thio-Michael addition of the omega chain end to the acrylamide.

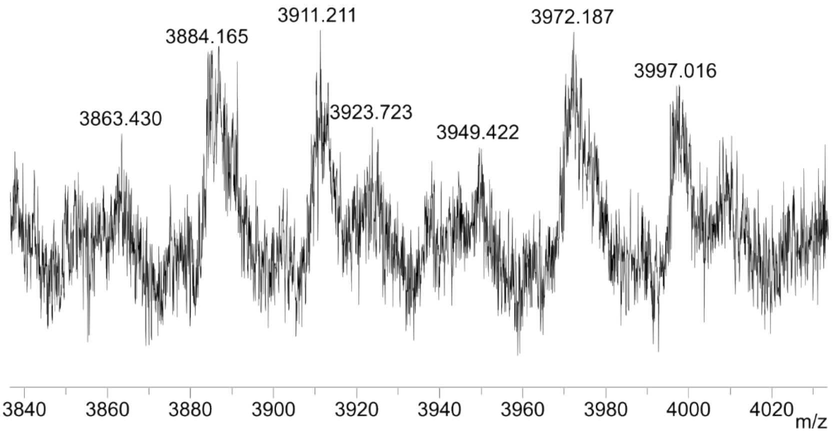

Figure S23. MALDI-TOF of PVAc modified by treatment of hexylamine and *N*-benzyl acrylamide. Peaks from centre of second distribution. We could not assign these masses to any expected structures, they could be from disulfides or other dimers.

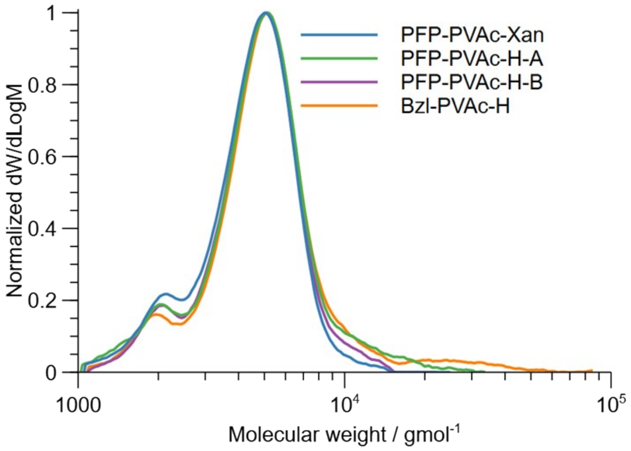

Figure S24. Molecular weight distributions of PVAc from SEC in DMF, calculated against PMMA standards.

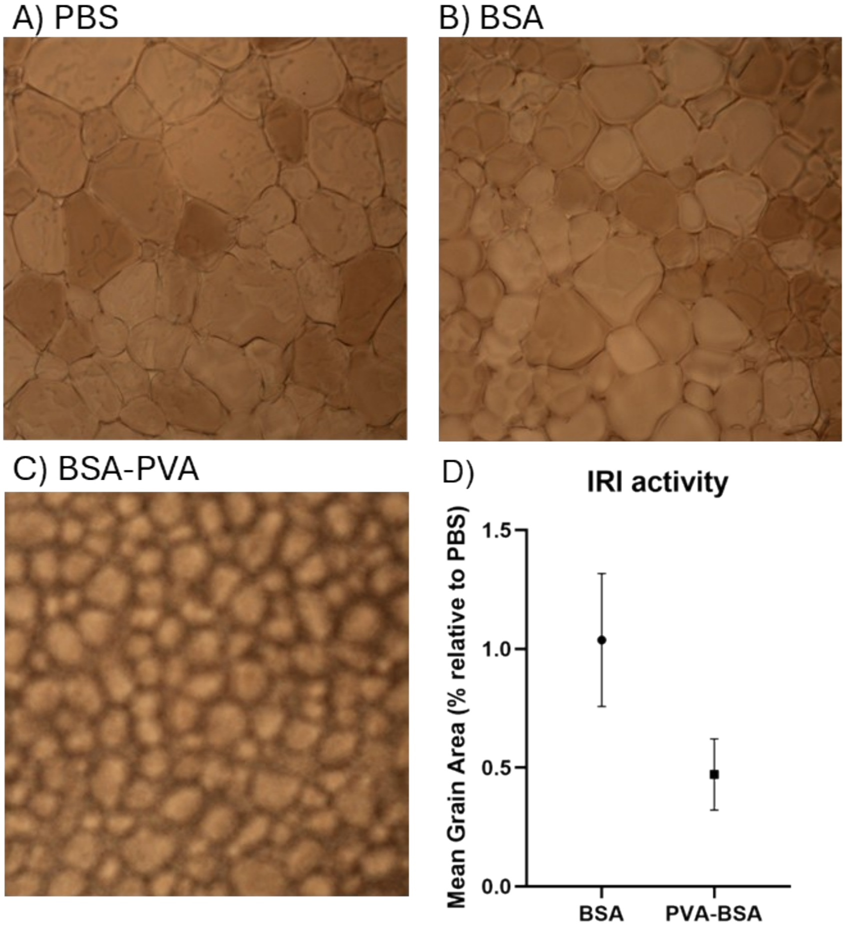

Figure S25. Ice recrystallisation inhibition activity of PVA/BSA conjugates. Light cryomicroscopy images from 'splat' assays of A) phosphate buffered saline; B) BSA in PBS; C) BSA-PVA fraction from FPLC in PBS; D) Comparison of IRI activity of BSA to BSA-PVA.

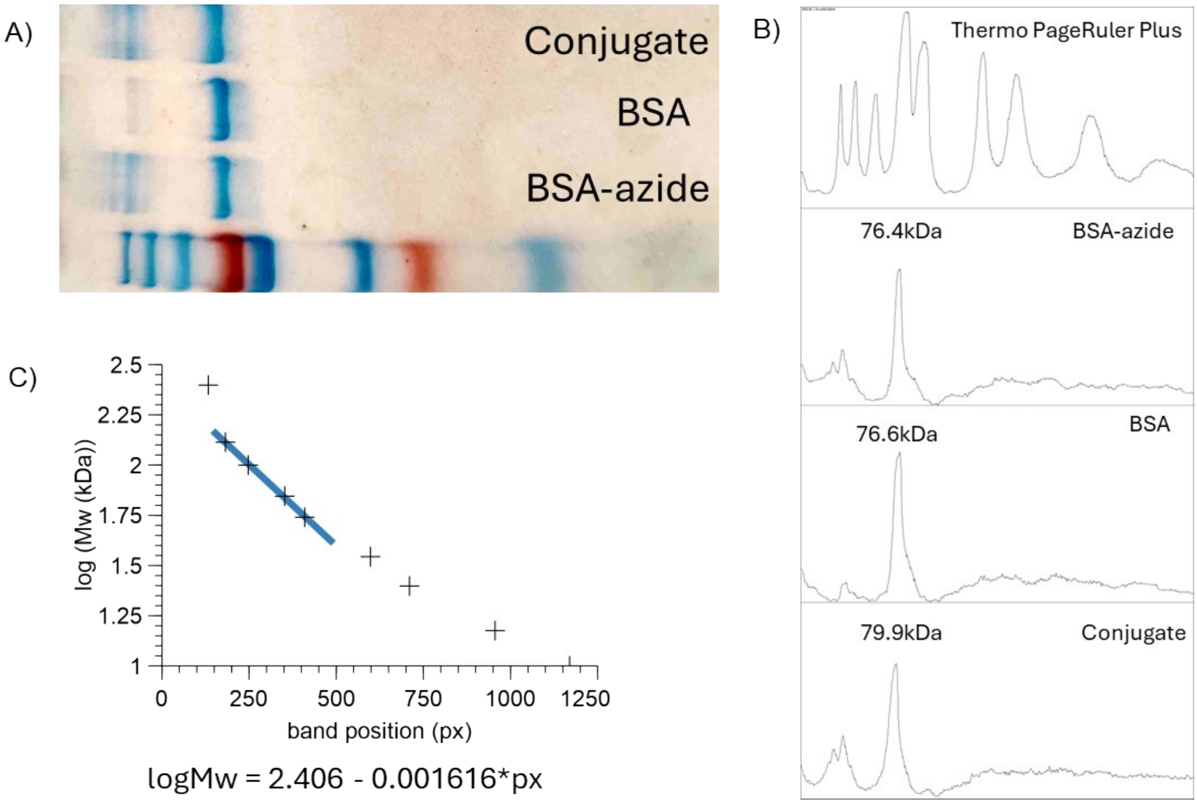

Figure S26. SDS-PAGE of BSA-N_3, DBCO-PVA conjugation (A). ImageJ gel tool was used to plot the intensity of each lane, after converting the image to monochrome (B). Pixel positions of proteins from Thermo PageRuler Plus were used to produce a calibration plot (C), and produce estimated molecular weights for BSA-azide, BSA and BSA-PVA.

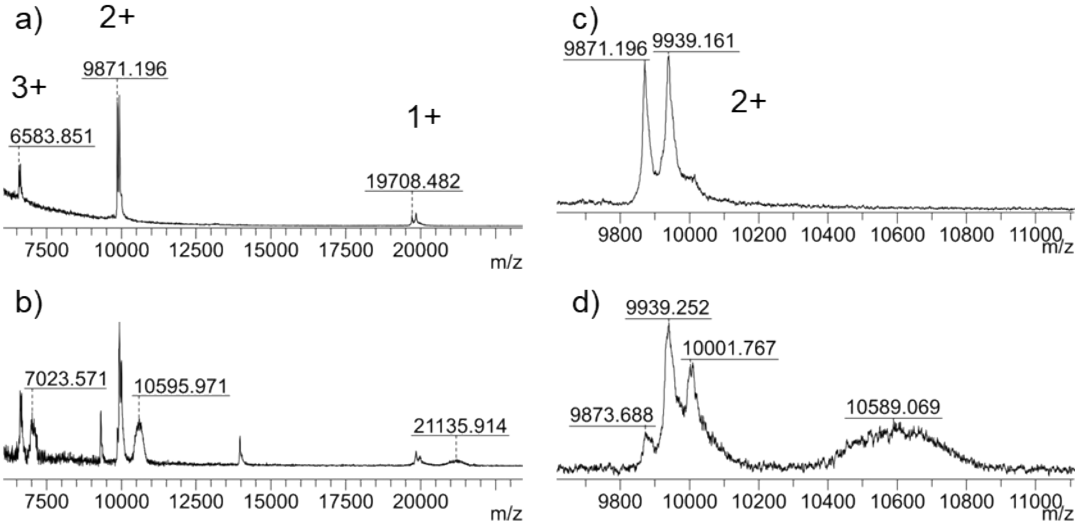

Figure S27. MALDI-TOF mass spectra of snap tag protein (A and C) compared to a mixture of snap tag protein and BG-PVA_{23} (B and D). Modification of the protein with PVA can be seen in three charge states. Since the signal to noise of the 2+ charge state was the highest we have shown that here. For SNAP tag protein alone, the unmodified form (9871.2) and the form

reduced by DTT (9939.2) can be seen. After the conjugation, the relative amount of unmodified form is greatly reduced.

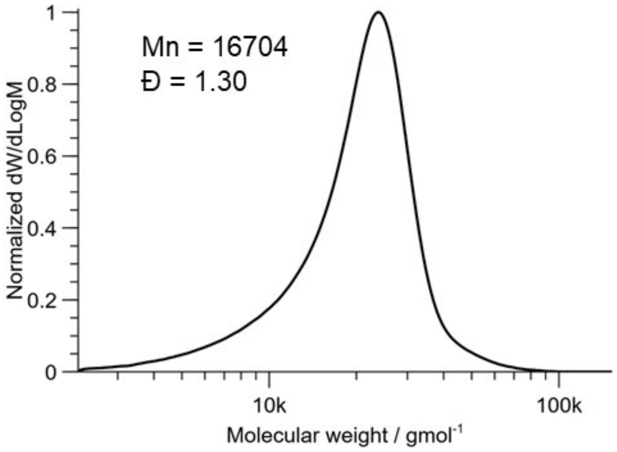

Figure S28. Molecular weight distribution of PFP-PVAc$_{110}$-Xan from SEC in DMF using PMMA standards.

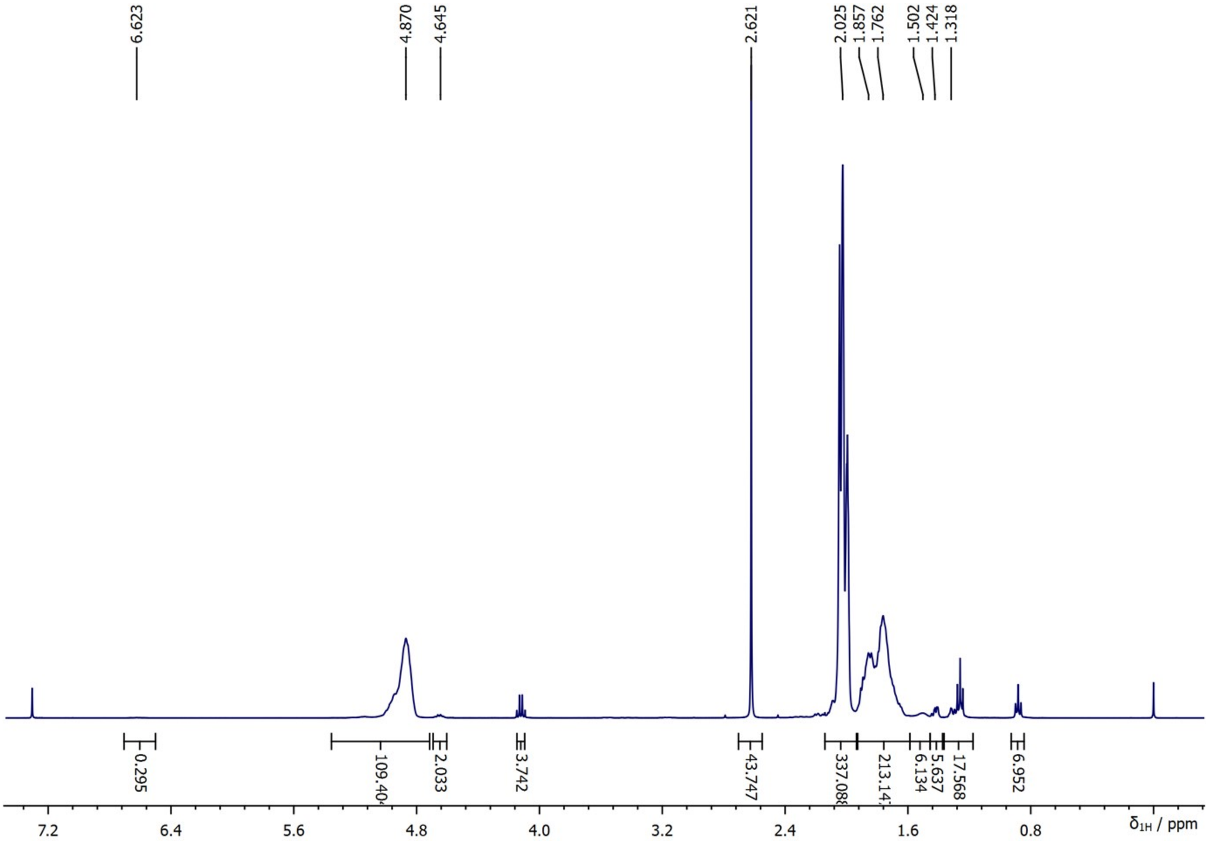

Figure S29. ^{1}H NMR of PFP-PVAc$_{110}$-Xan following precipitation. ^{1}H NMR (400 MHz, $CDCl_3$): δ 6.62 (1H, C(OAc)H-Xan), δ 4.87 (109H, backbone C(OAc)H), δ 4.65 (2H, xanthate CH_2), δ 2.03 (337H, $COCH_3$), 1.76 (213H, backbone CH_2), 1.5 – 1.2 (9H, xanthate CH_3 and R group $C(CH_3)_2$ + pentane)

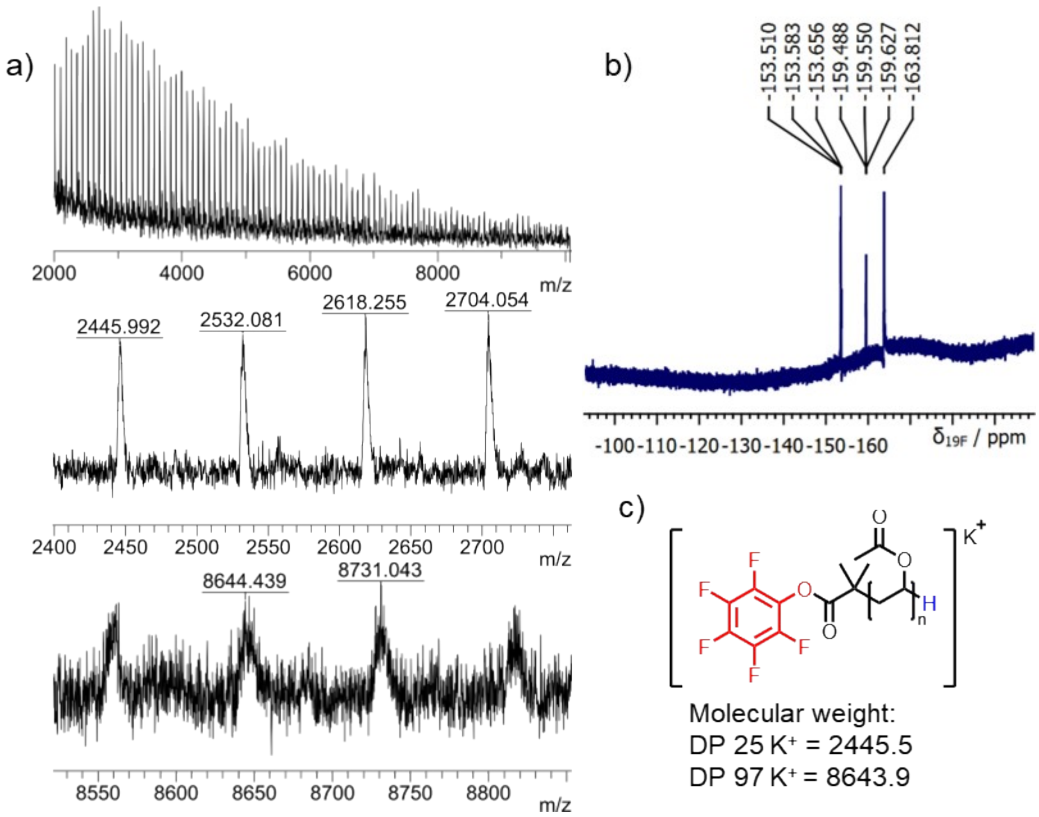

Figure S30. (a) MALDI-TOF of PFP-PVAc$_{110}$-H. The xanthate is removed for both low and high molecular weight chains. Note that in these conditions (DCTB, THF, KTFA) any mass spectra of PVAc polymers larger than 5 kDa were not reflective of the molecular weight distribution measured by SEC. (B) ^{19}F NMR spectrum of the reaction mixture following xanthate removal, showed no hydrolysis of the PFP ester after 24 hours; (C) Structure and predicted average molecular weights of PFP-PVAc-H.

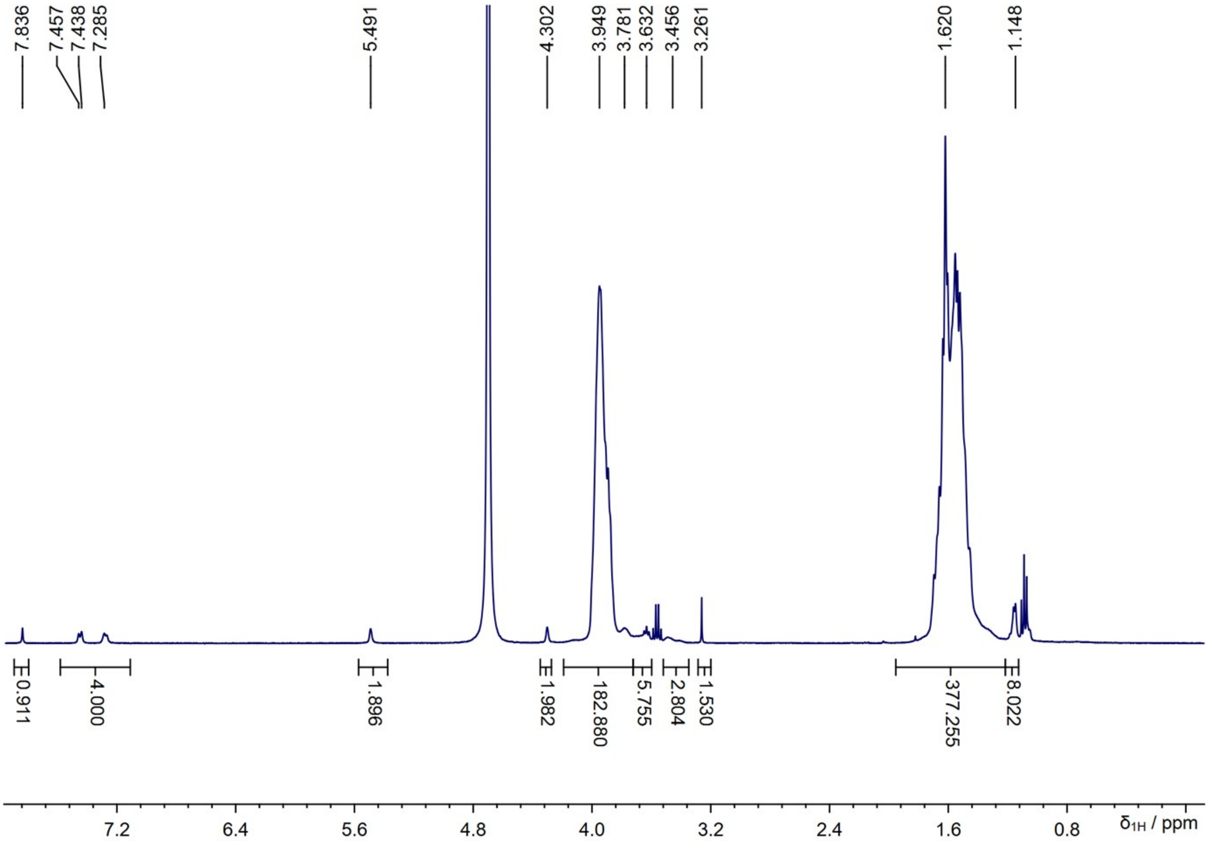

Figure S31. ^{1}H NMR spectrum of BG-PVA$_{110}$. ^{1}H NMR (400 MHz, D_2O): δ 7.86 (1H, BG N=C**H**NH), δ 7.46 – 7.29 (4H, BG benzyl CH), δ 5.91 (2H, O^{6}BG CH_2), δ 4.30 (2H, BG NHC**H**$_2$), 3.95 (182H, backbone C**H**OH), δ 1.62 (377H, backbone CH_2), δ 1.15 (6H R group $C(CH_3)_2$)

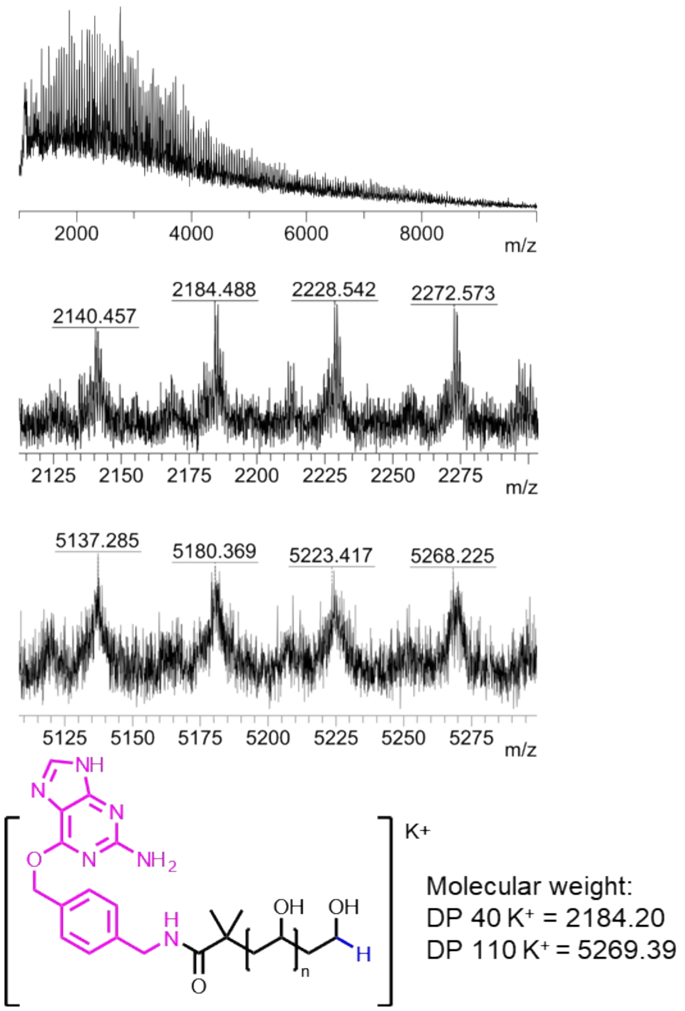

Figure S32. MALDI-TOF mass spectrum of BG-PVA_{110}
